# Altered fluvial patterns in North China indicate rapid climate change linked to the Permian-Triassic mass extinction

**DOI:** 10.1038/s41598-019-53321-z

**Published:** 2019-11-14

**Authors:** Zhicai Zhu, Yongqing Liu, Hongwei Kuang, Michael J. Benton, Andrew J. Newell, Huan Xu, Wei An, Shu’an Ji, Shichao Xu, Nan Peng, Qingguo Zhai

**Affiliations:** 1 Institute of Geology, Chinese Academy of Geological Sciences, Beijing, 100037 China; 2 School of Earth Sciences, University of Bristol, Bristol, BS8 1RJ UK; 3 British Geological Survey, Maclean Building, Wallingford, OX10 8BB UK; 4 College of Earth Sciences, East China University of Science and Technology, Nanchang, 330013 Jiangxi China; 5 Institute of Electronics, Chinese Academy of Sciences, Suzhou, 215123 Jiangsu Province China; 6 Shanxi Museum of Geology, Taiyuan, 030024 China

**Keywords:** Palaeoclimate, Environmental impact, Stratigraphy, Palaeontology, Sedimentology

## Abstract

The causes of the severest crisis in the history of life around the Permian-Triassic boundary (PTB) remain controversial. Here we report that the latest Permian alluvial plains in Shanxi, North China, went through a rapid transition from meandering rivers to braided rivers and aeolian systems. Soil carbonate carbon isotope (δ^13^C), oxygen isotope (δ^18^O), and geochemical signatures of weathering intensity reveal a consistent pattern of deteriorating environments (cool, arid, and anoxic conditions) and climate fluctuations across the PTB. The synchronous ecological collapse is confirmed by a dramatic reduction or disappearance of dominant plants, tetrapods and invertebrates and a bloom of microbially-induced sedimentary structures. A similar rapid switch in fluvial style is seen worldwide (e.g. Karoo Basin, Russia, Australia) in terrestrial boundary sequences, all of which may be considered against a background of global marine regression. The synchronous global expansion of alluvial fans and high-energy braided streams is a response to abrupt climate change associated with aridity, hypoxia, acid rain, and mass wasting. Where neighbouring uplands were not uplifting or basins subsiding, alluvial fans are absent, but in these areas the climate change is evidenced by the disruption of pedogenesis.

## Introduction

The severest ecological crisis in Earth history, the Permian-Triassic mass extinction (PTME), occurred 252 Ma and killed over 90% of marine species and about 70% of continental vertebrate families^1,2^. The driver of the event has been much debated, but the widely accepted killing model^2–7^ which involves massive volcanic eruptions in Siberia and associated emissions of greenhouse gases causing warming of land and oceans, stagnation and ocean floor anoxia, associated with acid rain that stripped the landscape of plants and soils and acidified the oceans. The sharp warming pulse of some 15 °C drove life from the tropics, loss of forests and soils deprived land life of food, and acidification of the oceans caused stress for calcifying marine organisms^8–12^.

The mass wasting of the land surface at this time has been controversial. Evidence for this process was first presented^13^ from the Permian-Triassic red bed successions of European Russia, where systems of predominantly cyclical, climate-driven, fine-grained deposits of lakes and meandering streams were supplanted by massive conglomerates deriving from huge alluvial fans up to 900 km long. The massive conglomerates were taken as marking the Permian-Triassic boundary (PTB), and so coinciding with the extinction crisis; this was confirmed by biostratigraphic and magnetostratigraphic work^13^. But what was the cause? Russian geologists had interpreted the sedimentary switch as evidence for the renewal of regional tectonics, and uplift of the Ural Mountains, from which the sediment was derived. They had also considered the idea that the sudden switch from meandering to braided streams indicated increased rainfall, but all evidence indicated increasing aridity across the boundary. Newell and colleagues^13^ showed there was no independent evidence for tectonic activity in the Urals, and so explained the sudden influx of masses of sediment as a result of the reduction of vegetation that had long been recognized in the subsequent ‘coal gap’, the 10 Myr time span in the Early and Middle Triassic^14,15^ when no coal, forests, or trees existed, presumably as a result of the devastating effects of the extinction.

**Figure 1:**
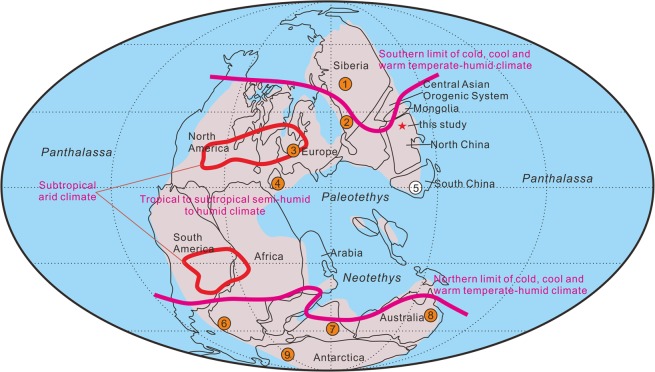
Palaeogeographic map and climate zones of the Permian-Triassic Pangea and locations of typical terrestrial Permian-Triassic sections (modified from Benton and Newell^10^). (1) West Siberian/Kuznetsk Basin. (2) Precaspian/Urals foreland basin/Russian Platform. (3) Central European Basin. (4) Iberian Basin. (5) South China. (6) Karoo Basin, South Africa. (7) Satpura/Raniganj basins, central India. (8) Bowen Basin, western Australia. (9) Victoria Land and the central Transantarctic Mountains, Antarctica. The numbered circles with orange for presence of alluvial fans and blank for no alluvial fans. Z.C.Z created this figure using CorelDRAW14.

The sudden switch from meandering to braided rivers at the PTB was reported independently^16^ from the Karoo Basin in South Africa, and the same explanation given. In a review^10^, similar sedimentary switches from low- to high-energy systems were noted also in Australia, Spain and possibly China (Fig. 1). The Chinese records to date were limited, however. Higher proportions of brecciated volcanic materials are common at the PTB in various terrestrial sections in South China^17^. These correspond to a colour shift from olive, grey or multi-coloured sandstones and mudstones of the coal-bearing uppermost Permian to maroon and purple interbedded sandstones and mudstones of the Early Triassic^10^. The earliest Triassic sediments are characterized by frequently occurring scours, poorly sorted syngenetic breccias and calcareous siltstone nodules, suggesting a dramatic collapse of soil systems near the PTB in South China^10^.

Mass wasting is corroborated from studies of shallow marine settings, where there were increases in soil-derived biomarkers indicating enhanced rates of weathering and erosion^18^. A sharply increased terrestrial sediment flux to marine depositional systems worldwide at the PTB is shown by increases in the bulk sediment accumulation rate, together with more clay-rich compositions^19,20^.

However, there is no such major shift in grain size across the PTB in the extensive sections of western Guizhou and eastern Yunnan^21–24^ and Xinjiang^25^, and this could be taken as evidence that the coarse-grained sediments were there, but have subsequently been lost (unlikely in so many well-preserved sections), or that coarse-grained material was not available in the eroding catchment area, or that these sections were distal from a proximal belt of coarse-grained deposition, or that the sedimentary shift had not occurred at these geographically widespread sites.

Here we explore new terrestrial PTB sections in Shanxi, North China, which confirm the environmental transition from meandering to braided rivers and the development of desert-like aeolian conditions in the earliest Triassic. The deterioration and fluctuation of the palaeoclimate are confirmed by δ^13^C, δ^18^O, and geochemical proxies of weathering intensity, while the ecological collapse is confirmed by a dramatic reduction or disappearance of dominant plants, tetrapods and invertebrates and a bloom of microbially-induced sedimentary structures (MISS)^26^.

## Results

### Geological setting

**Figure 2:**
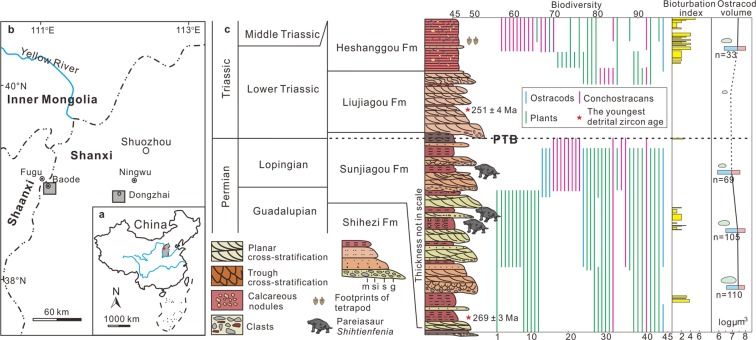
Maps, generalized columnar sections, and biotic variations. (**a**) Map of China on which the Shanxi Province is in grey and the generalized location is marked with a star. (**b**) Geographic map of northern Shanxi Province and its adjacent area on which the locations of Baode and Dongzhai are marked with a grey box. (**c**) Integrated stratigraphic column from Guadalupian to Early Triassic in North China (modified from Chu *et al*.^26^). Fossil data come from Supplementary Tables S1 and S2. The data on ostracod sizes are in Supplementary Table S3. Abbreviations: m, mudstone; si, siltstone; s, sandstone; g, conglomerate; Fm, Formation; Footprints and pareiasaur schematically reconstructed.

Terrestrial Permian-Triassic strata are widely distributed in North China, and show similar terrestrial fluvial-lacustrine sedimentary facies through the Permian-Triassic transition, against a background of regional regression (Fig. 2). The sedimentary succession through the Upper Permian and Lower Triassic in North China generally comprises, in ascending order, the Guadalupian-Lopingian (Middle-Late Permian) Sunjiagou, Early Triassic Liujiagou, and late Early Triassic Heshanggou formations (Fig. 2). The age of the Sunjiagou Formation is confirmed by finds of the typical Lopingian plant *Ullmannia* in its upper part in the Dayulin and Sugou sections (Supplementary Tables S1–S3)^26^. The PTB is identified at a horizon about 20 m below the top of the Sunjiagou Formation by the occurrence of the *Lundbladispora-Aratrisporites-Taeniaesporites* assemblage^26^, which represents typical earliest Triassic palynomorphs^27^. The PTB is confirmed by the last appearance of bioturbation (higher than the last appearance of the pareiasaur *Shihtienfenia*^28^) and a synchronous negative excursion of δ^13^C, δ^18^O and geochemical proxies at a horizon about 15 m below the top of Sunjiagou Formation. The mixed fluvial-aeolian Liujiagou Formation is characterized by the abundance of cross-bedding and rare fossils^27^. Only rare species (Fig. 2) like the plant *Pleuromeia* (a typical Early Triassic element^29^) were found in the upper part of the Liujiagou Formation. Moreover, a variety of MISS, most of which were formed after the mass extinction, were recognized in the Liujiagou Formation^26^. The late Early Triassic Heshanggou Formation mainly consists of red siltstones and mudstones with abundant and increasingly diverse trace fossils (Fig. 2). The stratigraphy of two well-exposed outcrops in northern Shanxi Province were measured at high resolution, using the newly found pareiasaur *Shihtienfenia*^28^ assemblage in the Baode section as biostratigraphic evidence to identify the age as latest Permian.

### Sedimentology

**Figure 3:**
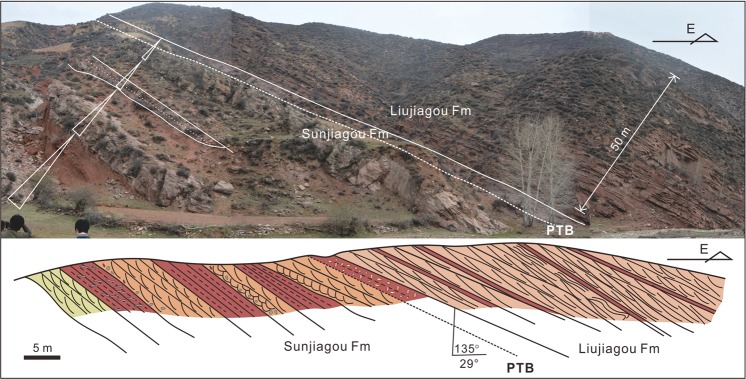
Stratigraphic overview showing the contact between the Sunjiagou and Liujiagou formations. White triangles denote upward fining cycles in the Sunjiagou Formation. Dotted line denotes base of the PTB interval. GPS: 38° 45′ 59.60″ N, 112° 04′ 50.222″ E. Abbreviations: Fm, Formation; PTB, Permian-Triassic boundary; Z.C.Z created this figure using CorelDRAW14. Photo credit: Y.Q.L.

**Figure 4:**
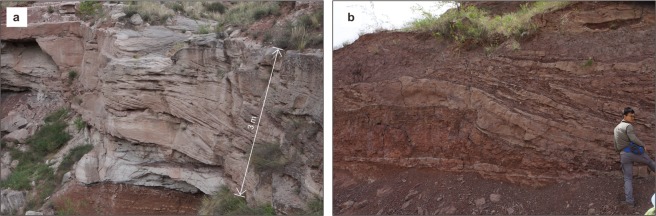
Typical features of the Sunjiagou Formation. (**a**) Amalgamated cross-bedded sandstone deposited in the lower parts of channel point bars overlying a sharp erosion surface on overbank mudstones. (**b**) Upper point bar deposits comprising laterally accreted beds of sandstone and mudstone. Photo credit: Y.Q.L.

Three distinct facies associations were identified. Below the PTB (Fig. 3), lithostratigraphic associations in the Sunjiagou Formation are characterized by sequences of light grey greenish pebble-bearing coarse sandstones up to 25 m thick interbedded with sequences of fine sandstone and dark red mudstone up to 20 m thick. Each sandstone sequence typically overlies a sharp, undulating erosion surface cut into dark red mudstone (Figs 3 and 4a). Internally the sequences comprise multiple storeys bounded by low-angle erosion surfaces, often overlain by reworked dark red mudstone clasts, and thin mudstone interbeds. Storeys are composed mainly of metre to decimetre thick sets of planar–tabular and trough cross-bedded sandstone, horizontal lamination and asymmetrical ripple cross-laminated sandstone. These erosively amalgamated units of cross-bedded sandstone represent the deposits of bars within a fluvial channel or channel belt where there were multiple episodes of deposition and erosion. A predominance of point bar deposition with a meandering channel belt is suggested by the occasional preservation of upper bar deposits which show well-developed lateral accretion surfaces composed of alternating inclined beds of dark red mudstone and fine-grained sandstone with climbing ripple cross lamination or horizontal lamination (Fig. 4b). These upper bar deposits are diagnostically important, but have a relatively low preservation potential within a fluvial belt where channel migration and reworking is rapid relative to tectonic subsidence. The dark red mudstones and thin fine-grained sandstones represent the deposits of floodplains adjacent to meandering channel belts. Calcareous nodules occasionally occur scattered or in layers within the mudstones and probably represent calcretes formed within floodplain soil horizons subject to extended periods of subaerial exposure (Fig. 4a). The pareiasaur *Shihtienfenia* fossils and the PTB itself are found within floodplain deposits characterized by thick dark red mudstone or pebbly mudstone with a few calcareous nodules.

**Figure 5:**
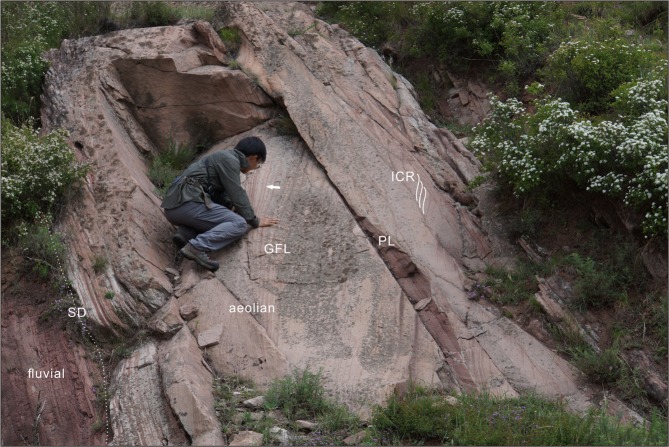
Aeolian dune and wind ripple deposits overlying a sand-drift surface on fluvial sandstones within the Liujiagou Formation. The white arrow points to the aeolian pin stripe lamination. Abbreviations: SD, sand-drift surface; PL, planebed lamination; ICR, inverse climbing-ripple; GFL, grainfall lamination. Photo credit: Y.Q.L.

The facies association above the PTB is dramatically different. Mudstone layers are poorly developed in the Liujiagou Formation, and there is a much higher proportion of sandstones, which shows evidence for both fluvial and aeolian depositional environments (Fig. 5). Fluvial sandstones show trough and planar cross-bedding ranging from low-angle to high-angle. Irregular scour surfaces are common and are locally overlain by maroon mudrock clasts. The well-developed thick and coarse interchannel sequences in the lowermost Triassic Liujiagou Formation suggest an overall higher rate of sedimentation^30^ compared with the Sunjiagou Formation. Aeolian sandstones sharply overlie fluvial units on sand-drift surfaces^31^ and show characteristic sedimentary structures such as pin stripe lamination^32^, grainfall laminations developed on dune slip faces^33^, and wind ripple lamination^34^ which may have formed on low-relief aeolian sand sheets or around the flanks of dunes (Fig. 5). The complex lateral and vertical stacking of fluvial and aeolian facies indicates that the lowermost Triassic Liujiagou Formation was deposited in an environment where ephemeral sandy braided rivers interacted and alternated with aeolian processes. Comparable mixed fluvial-aeolian deposits have been identified in the Jurassic Tianchihe Formation of Ningwu-Jingle Basin, North China^35^ and in the Early Triassic of the western European basins^36,37^.

The late Early Triassic Heshanggou Formation is dominated by shallow lacustrine deposits, characterized by cycles of siltstones and/or mudstones interbedded with a few thin fine-medium grained sandstone layers. Bioturbation in the Heshanggou Formation becomes stronger from the bottom to top, suggesting biological recovery after the extinction near the PTB^26^ (Fig. 2c). Diverse trace fossils and the bioturbation index^26,38^ in the Sunjiagou and Heshanggou formations are summarized in Fig. 2 (Supplementary Fig. S1).

### Geochronology

Two detrital zircons yield youngest ages of 269 ± 3 Ma and 251 ± 4 Ma respectively, from the Sunjiagou Formation (sample 2015BDSJG-1, Supplementary Table S6) and the Liujiagou Formation (sample 2015BDLJG-1, Supplementary Table S6). They constrain the age of the Sunjiagou Formation as Guadalupian–Lopingian and the Liujiagou Formation as lowermost Triassic. Combined with previous biostratigraphic data and the mass extinction at the uppermost Sunjiagou Formation, as well as the sedimentary environmental transition from the Sunjiagou Formation to Liujiagou Formation, these dates convincingly constrain the terrestrial PTB in North China. Moreover, the detrital zircon age results from the Sunjiagou and Liujiagou formations in this study show nearly the same characteristics of age groups and peaks (Supplementary Fig. S2). Combined with similar age populations of detrital zircons from the Late Permian-Early Triassic strata nearby^39,40^, it is therefore compelling to infer that the Sunjiagou and Liujiagou formations in northern Shanxi, North China share the same source area, originating from the Yinshan–Yanshan Orogenic Belt and Northern Qinling Orogen.

### Geochemical evidence

Diagenetic alteration of the elemental and isotopic elements in ancient carbonates and mudstones can obliterate primary depositional trends and therefore these require an adequate assessment. The Mn/Sr ratio has been regarded as a sensitive indicator of diagenetic alteration in carbonates^41–44^ and, while Derry *et al*.^42^ and Kaufman *et al*.^43,44^ suggested that samples with Mn/Sr < 2–3 were unaltered, later work has suggested that samples with values as high as 10 still produced reliable carbon isotope signatures^45–47^. The Mn/Sr ratio yields values of 0.61–6.48 in this study (Supplementary Table S5) and most of them were <3, which suggest these samples were nearly unaltered, so they preserve primary depositional characteristics. Moreover, the carbon and oxygen isotope cross-plot (Supplementary Fig. S3) shows no obvious positive linear relationship between δ^18^O and δ^13^C values, and data points are relatively discrete, indicating that these elements are basically not affected by diagenesis, and the resulting isotope data are reliable^47–51^. Previous research^e.g.^ ^47,52^ shows that oxygen isotopes may have undergone strong diagenesis when the sample δ^18^O < −10‰. However, carbon isotopes are less susceptible to diagenesis than oxygen isotopes^53^ and in some cases where the formation of a diagenetic change occurs, the original δ^13^C may still be well preserved^54,55^. Therefore, in current research, sometimes a δ^13^C value can also be valid when δ^18^O < −10‰^56^. The Ti/Al ratio is a useful indicator in discrimination of source area differences and has been applied to rocks spanning from the Precambrian through to the Cenozoic^57–60^. In this study, the value of most Ti/Al ratios is 0.4–0.6 (Supplementary Table S5) except for a few abnormal data, and the relatively stable Ti/Al ratio suggests an unchanging source area for the sediments comprising the Sunjiagou and Liujiagou formations.

**Figure 6:**
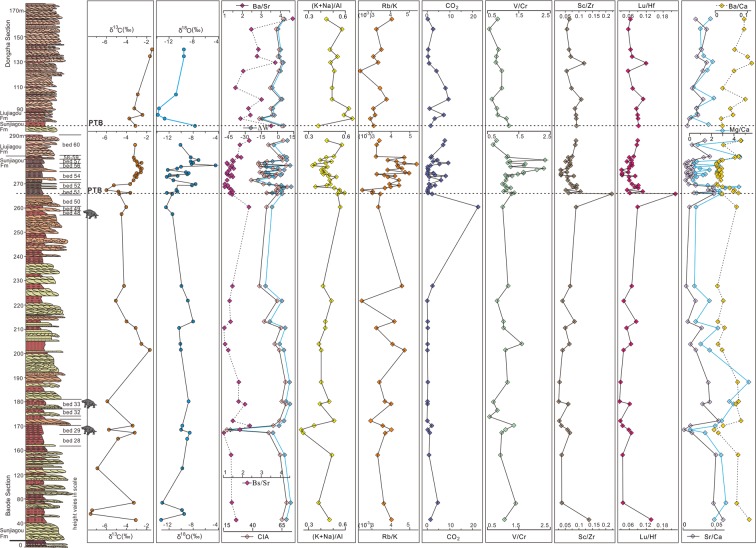
High-resolution δ^13^C, δ^18^O and geochemical proxy profiles in the latest Permian-Early Triassic strata from the measured section. Details are presented in Supplementary Tables S4 and S5. Abbreviations: Fm, Formation; PTB, Permian-Triassic boundary. Z.C.Z created this figure using CorelDRAW14. Footprints and pareiasaur body shape are schematically reconstructed.

The δ^13^C (Supplementary Table S4) profiles across the PTB range from −1‰ to −7.8‰ (VPDB) and −1.5‰ to −3.8‰ (VPDB) (Fig. 6) respectively. They exhibit a weak negative trend and stronger negative excursion at the three pareiasaur *Shihtienfenia* fossil spots and bed 51 (−3.7‰ to −6.0‰, VPDB) of the PTB interval. Values of δ^18^O (Supplementary Table S4) show consistent variations with δ^13^C (Fig. 6) and exhibit strong perturbation at the PTB interval (−4‰ to −12‰ at Baode and −7.8‰ to −13.9‰ at Dongzhai). Overall, the perturbation of δ^13^C and δ^18^O in this study show similar trends with other typical terrestrial (e.g. Karoo Basin^61^, East Europe^62^, Antarctica^63^, Xinjiang of northwestern China^25^; Fig. S4) and marine PTB sections (e.g. Meishan section of South China^25,64^) elsewhere in the world.

Geochemical proxies (Fig. 6) of weathering intensity including Ba/Sr^58,65^, CIA^65^, and its modified version ∆W^65^ show consistent negative excursions at the fossil assemblage zone and the PTB interval. Sensitive proxies of salinity change including Rb/K^66^ and (K + Na)/Al^65^ are relatively low (Fig. 6), whereas Rb/K values apparently increase at the fossil spots and the PTB interval. Values of (K + Na)/Al and Al/Si^66^ (Supplementary Table S5) indicate a relatively low palaeotemperature at the fossil spots and the PTB interval. Mg/Ca, Sr/Ca and Ba/Ca^67,68^ (Fig. 6) consistently show negative excursions at the fossil assemblage zone and the PTB interval while Sc/Zr and Lu/Hf^67^ show strong negative excursions at these points.

## Discussion

Palaeontological and geochemical evidence demonstrate that the PTB in the Shanxi red beds is at a horizon about 15 m below the top of Sunjiagou Formation (Figs 3 and 4), and the detrital zicon ages from the Sunjiagou and Liujiagou formations support this scenario (Supplementary Table S6). The synchronous dramatic negative excursion in δ^13^C and δ^18^O in the uppermost Sunjiagou Formation provide reliable evidence for reduced weathering, coolness, aridification, and anoxia^69^ (Fig. 6). Increasingly high negative values of δ^13^C and δ^18^O in the latest Permian in North China (Fig. 6) reveal the intensified coolness and aridity at that time. In particular, their remarkable shift indicates multiple rapid fluctuations of palaeoclimate, and the sharp negative excursion of δ^13^C in bed 51 of the Baode Section was coeval with the last occurrence of bioturbation before the end of the Permian. The sharp negative isotopic excursions were probably a result of dramatic climate perturbations on land and a decrease in vegetation density, which was a response to the ongoing cooling and aridification^70,71^. A similar shift and negative excursion of δ^13^C and δ^18^O around the terrestrial PTB were also confirmed in the Karoo Basin in South Africa^18^ and elsewhere (Supplementary Fig. S4). Moreover, the strong positive excursion of Sc/Zr and Lu/Hf also supports an abrupt arid palaeoclimate change^67^.

The negative excursion of weathering intensity proxies such as Ba/Sr, CIA, ∆W, Mg/Ca, Sr/Ca and Ba/Ca support the reduced weathering in the latest Permian, especially at the fossil horizons and the PTB interval, but increasingly intense weathering in the Early Triassic. The apparent increase of Rb/K at the fossil horizons and the PTB interval indicate a brief episode of drier and more arid conditions^72^. Mg/Ca, Sr/Ca and Ba/Ca (Fig. 6) are important proxies^67,68^ to distinguish the palaeoclimate of weathering intensity and palaeotemperature and they have been successfully applied to the monsoon of East Asian^68^.

The relatively cool palaeotemperature in the PTB interval and fossil horizons were proved by δ^18^O, (K + Na)/Al, Al/Si, Mg/Ca, Sr/Ca and Ba/Ca (Fig. 6 and Supplementary Table S5), which show synchronous negative shifts at these important times. Although palaeotemperatures calculated from the main elements might be affected by local conditions and changes of sediment provenance, they may play an important role in identifying palaeoclimate fluctuations when sampling is appropriate and combined with other indicators^73^. Our study indicates a relatively cool temperature across the PTB, which was supported by some previous studies^74–77^ though it is different from most views that indicate a rapid increase in palaeotemperature across the PTB. However, in models for the outcomes of a massive volcanic eruption, such as that of the Siberian Traps, release of massive volumes sulphur dioxide when mixed with atmospheric water may produce a transient cooling phase before the warming, driven by CO_2_, methane and water vapour. Such cooling can be localised around the volcanic source, or can spread worldwide and last for 1–2 years^78^. Whether the conflicting findings of either global warming or cooling following the PTB eruptions can be explained by these differing consequences of the eruption, perhaps acting in sequence, or whether these differing temperature changes reflect latitudinal or regional regional effects cannot at this stage be determined.

The atmospheric *p*CO_2_ estimated from δ^13^C values^66,70^ and whole-rock CO_2_ values^79^ consistently show an abrupt and remarkable increase at the fossil horizons and the PTB interval, which was an important part of deteriorating palaeoclimate and could be a crucial factor in biotic extinction^36,80^. The negative δ^13^C shift and sharp increase of whole-rock CO_2_ are direct reflections of changes of atmospheric *p*CO_2_, which is supported by previous studies focusing on the abnormal occurrence of contemporaneous significant negative δ^13^C both in the ocean and on land^81^. They might have been caused by a significant input of methane into the atmosphere^82^ at the end of the Permian. The Siberian traps basaltic eruptions^6^ and the closure of the eastern segment of the Palaeo-Asian Ocean^83^ could have contributed to the input of methane at the PTB. Moreover, values of V/Cr > 2 are considered to represent anoxic depositional conditions^79^, which occasionally occurred in the PTB interval.

Above all, the fluvial environmental transition from meandering to braided river-aeolian, δ^13^C and δ^18^O, as well as geochemical proxies, reveal a consistent pattern of deteriorating environments (reduced weathering, cool, arid, and anoxic conditions) and climate fluctuations before and through the PTB. Nevertheless, the persisting uplifting tectonics in northern North China, as a response to the final closure of the Palaeo-Asian Ocean and collision between the Mongolian arc and North China Craton along the Solonker Suture Zone^83^, may contribute to the influx of masses of sediment through the Permian-Triassic transition. Moreover, matching the increasingly deteriorating environmental change across the PTB in North China, studies of the marine Permian-Triassic throughout the world show intensified chemical and physical weathering^66,84–86^ and anoxic conditions^7,66,87,88^ across the PTB.

It is reasonable to assume that the switch in fluvial style is largely linked to global climatic change when we combine the simultaneous environmental changes (e.g. arid, cool, and anoxic conditions) and mass extinction across the PTB. The abrupt environmental shocks (e.g. hypoxia, aridity, acid rain and wasting, etc.) were probably the main causes of the PTME on land^7–11^. The increasingly dry and deteriorating ecological environment resulting from warming and acid rain caused nearly worldwide mass wasting. The remarkable change in fluvial pattern at the PTB in Shanxi confirms results found earlier in Russia^13^ and South Africa^16^, and coincides with the increasingly arid palaeoenvironment throughout the terrestrial PTB in North China. In particular, the well-preserved aeolian deposits in the Liujiagou Formation are a critical sedimentological marker of aridity, highly-erodible land surfaces possibly related to reduced vegetation cover and sufficient wind energy to entrain and transport sediment. Elevated aeolian activity has been noted also in the Lower Triassic of the western German Basin^36^. Similar radical turnovers in fluvial style across the terrestrial PTB were also recognized in the Permian-Triassic red beds of eastern Europe^13^, the Karoo Basin^16^, and Australia^80^.

The distribution of climate zones near the PTB (Fig. 1) shows polar zones of cold, cool, and temperate humid climates, and a broad equatorial belt of tropical to subtropical semi-humid to humid climates, extending from Canada, Russia, and North China to central Africa, and the southern margin of Tethys^10,37,89^. Two mid-continental areas of subtropical arid climates occur over the eastern United States and western and central Europe, in the north, and central South America-Africa in the south. As noted before^10^, the most extreme changes occur in high-latitude, cool temperate settings (Australia, Antarctica, Siberia) and in the ever-wet coastal tropics (e.g. India, South China), where peat-forming swamps disappeared suddenly at the end of the Permian, initiating the coal gap^15^ of the Early and Middle Triassic. In tropical, semi-humid basins such as east European Russia^13^ and South Africa^16^, brown overbank mudstones with plant remains in the late Permian are replaced by highly oxidised red mudstones in the Early Triassic, often reworked into coarse braided river deposits. Our new evidence from North China is located at a similar latitude to the Russian red beds with PTB conglomerates, differing considerably from the successions 1200 km south, in South China.

Why do some terrestrial sections show the dramatic shift from meandering streams and lakes to arid alluvial fans-braided streams at the PTB^10,13,16,80,90^, and others do not^21–25^? It is not purely a question of palaeogeographic or palaeoclimatic locale, as noted. The PTB sections in which massive conglomerates are absent, for example in South China, India and Antarctica lie in equatorial, subtropical arid, hot, and humid climate belts (Fig. 1). One hypothesis is that the fan conglomerates were there but were lost through incomplete preservation. This might apply in some sections, but it is unlikely that such massive, coarse-grained units could be entirely removed from hundreds of sections in these regions. If the mass wasting model is correct, then the enhanced erosion following stripping of vegetation and increased aridity would be expressed in different ways in different sedimentary basins. Alluvial fan progradation can occur without tectonic uplift in the source area, and in such cases the progradation is largely controlled by a decrease in subsidence rates in the basin^91,92^. However, for substantial alluvial fans to develop as a result of plant and soil stripping requires a developing imbalance between source and receiving basinal areas, with tectonic uplift of upland areas round the basin, or subsidence of the basin. Perhaps at the PTB, in the face of worldwide mass wasting, as suggested by offshore records of terrestrial sediment flux^19^, those locations without conglomerates were simply in basins without surrounding mountainous source areas, or where the relative uplift-subsidence activity was inappropriate. In South China, for example, an abrupt shift in sedimentation is seen immediately following the disappearance of coal beds (beginning of the coal gap), with colour and grain-size changes in the sediments, together with indicators of a dramatic collapse of soil systems^10^. Besides, most dramatic shifts of fluvial deposits are distributed in areas of similar latitude. Comparison with the distribution of modern deserts along the equatorward margins of sub-tropical basins which are climatically sensitive^93^ may give some clues to the distribution of different terrestrial Permian-Triassic sections.

In this study, the terrestrial Permian-Triassic transition in North China is well constrained by multiple lines of evidence, namely sedimentology, carbon and oxygen isotopic results, geochemical proxies, and detrital zircon ages. In the near future, more integrated work on PTB sections in North China needs to be done in order to make a high-resolution regional stratigraphic comparison at regional and global scale.

## Methods

### δ^13^C and δ^18^O analysis

Forty-nine palaeosol carbonate samples were selected for δ^13^C and δ^18^O analysis. Bioturbation and ped structure and lack of primary laminae and beds are good indicators to judge the soil development. To avoid weathered surfaces and minimize the effects of diffusion and diagenesis^94,95^, each sample was collected at least ~50 cm below the section surface. Carbonate samples were converted to CO_2_ using dehydrated phosphoric acid under vacuum at 70 °C for 1 hour. Carbon and oxygen isotope ratios were measured by a MAT-253 mass spectrometer at the National Research Center for Geoanalysis, Chinese Academy of Geological Sciences (CAGS), Beijing. Isotope values were normalized to in-house standards by calibration against NBS-19 carbonate reference material. The measurement precision was < 0.1‰ in general and checked by NBS-19 (δ^18^O = −2.20‰, δ^13^C = + 1.95‰, PDB standard) and NBS-18 (δ^18^O = −23.2‰, δ^13^C = −5.1‰, PDB standard). All isotope ratios are given in ‰ relative to PDB. Carbon- and oxygen-isotope results are shown in Supplementary Table S4 and used to predict palaeotemperature^69,70^, weathering intensity^71^, atmospheric CO_2_^69^, and plant biomass^81^.

### Geochemical analysis

Fifty-seven mudstone samples were selected to conduct whole-rock major and trace element compositions analysis at the National Research Center for Geoanalysis, Chinese Academy of Geological Sciences (CAGS), Beijing. The analysis procedure is similar to Zhai *et al*.^96^ Major elements were analyzed by X-ray fluorescence (XRF), and analytical uncertainties were commonly better than 1% for all elements of these samples. Trace element concentrations were analyzed using an Agilent-7500a inductively coupled plasma mass spectrometer (ICP-MS). Sample preparation followed usual protocols. We dissolved about 50 mg of sample powder in an equal mixture of sub-boiling distilled HF and HNO_3_ in a Teflon digesting vessel (high-pressure bomb) retained on a hot-plate for 48 h. We then evaporated the dissolved sample until dry, refluxed it with 6 N HNO_3_, and re-heated it to incipient dryness. We then redissolved the samples in 2 ml of 3 N HNO_3_ in high-pressure bombs for an additional 24 h to ensure complete dissolution. After digestion, we diluted the samples with Milli-Q water (18.2 mega-ohm) to a final dilution factor of 2000. We used the rock reference materials AGV-2 (US Geological Survey) and GSR-1 (National Geological Standard Rreference Materials of China) to monitor the analytical accuracy and precision. We found that analytical accuracy, as indicated by relative differences between measured and recommended values, was better than 5% for most elements. We list the calculated results of the whole rocks and trace elements used in this study in Table S5.

### Geochemical proxies

Calculations of CIA^65^ and ∆W^65^ are,

$${\rm{CIA}}=100\,\ast \,{\rm{Al}}/({\rm{Al}}+{\rm{Ca}}+{\rm{K}}+{\rm{Na}})\,{\rm{and}}\,\Delta W={{\rm{CIA}}}_{{\rm{X}}}\mbox{--}{\rm{\mu }}\mathrm{CIA},$$

where each of the elemental concentrations is converted to moles^65^. μCIA represents the average value of CIA, CIA_X_ represents each value of the calculated CIA.

### Geochronology

Two medium sandstone samples from the Sunjiagou Formation (sample 2015BDSJG-1, Supplementary Table S6) and the Liujiagou Formation (sample 2015BDLJG-1, Supplementary Table S6) were collected for zircon U-Pb dating so as to constrain the depositional age. We separated zircon grains by conventional heavy liquid and magnetic techniques at the Special Laboratory of the Geological Team of Hebei Province, Langfang, China. In order to investigate the origin and structure of zircon, and select the target for U-Pb analysis, we obtained cathodoluminescence (CL) and reflected- and transmitted-light images. We generated CL images using a HITACHI S-3000 N scanning electron microscope fitted with a Gatan Chroma cathodoluminescence imaging system at the Institute of Geology, CAGS, Beijing, China. We undertook zircon U-Pb analyses using a laser-ablation-inductively coupled plasma-mass spectrometer (LA-ICP-MS) at the Institute of Mineral Resources, CAGS, Beijing, China. For each sandstone sample, we randomly selected 90 or 100 zircon grains for analysis, leaving out zircon grains with cracks or inclusions. We carried out laser sampling in an ESI NWR 193 nm laser ablation system, and acquired ion-signal intensisities with an AnlyitikJena PQMS Elite ICP-MS instrument. The analysis beam was ~25 μm in diameter, with 10 Hz repetition rate, and 4 J/cm^2^ of energy. The analytical procedures followed Hou *et al*.^97^. We then performed off-line raw data selection, integration of background and analyte signals, and time-drift correction and quantitative calibration for U-Pb dating using ICPMSDataCal^98^. We made age calculations and concordia diagrams using Isoplot/Ex ver. 3.0^99–107^. During the analysis, we analyzed the zircon standard GJ-1 to evaluate accuracy and precision. The results are listed in Supplementary Table S6. The errors for individual analyses are quoted at 1σ level, whereas the errors for weighted mean ages are quoted at 2σ (95% confidence level). For zircons younger than 1000 Ma, ^206^Pb/^238^U ages are used, but for zircons older than 1000 Ma, ^207^Pb/^206^Pb ages are used. ^206^Pb/^238^U ages >10%, >20% discordance or >5% reverse discordance are omitted from further consideration.

## Supplementary information

Supplementary information

## Data Availability

All data reported in this paper are available in the manuscript and supplementary materials.
