# Supplementary material for: Altered fluvial patterns in North China indicate rapid climate change linked to the Permian-Triassic mass extinction
Source: Sci Rep. 2019 Nov 14;9:16818. doi: 10.1038/s41598-019-53321-z (PMC6856103; doi:10.1038/s41598-019-53321-z)
Supplement: Supplementary file 1 — Supplementary information [file 41598_2019_53321_MOESM1_ESM.docx]

**Altered fluvial patterns in North China: Implications for drastic climate change related to the Permian-Triassic extinction**

Zhicai Zhu^[[1]](#footnote-1)^, Yongqing Liu^1^*, Hongwei Kuang^1^*, Michael J. Benton^2^, Andrew J. Newell^3^, Huan Xu^4^, Wei An^5^, Shu’an Ji^1^, Shichao Xu^6^, Nan Peng^1^ & Qingguo Zhai^1^

Fig. S1. **Bioturbation in the Sunjiagou and Heshanggou formations from northern Shanxi, North China.** (**a/b**) Bioturbation in the Sunjiagou Formation. (**c/d**) Bioturbation in the Heshanggou Formation. Z.C.Z created this figure using CorelDRAW14.


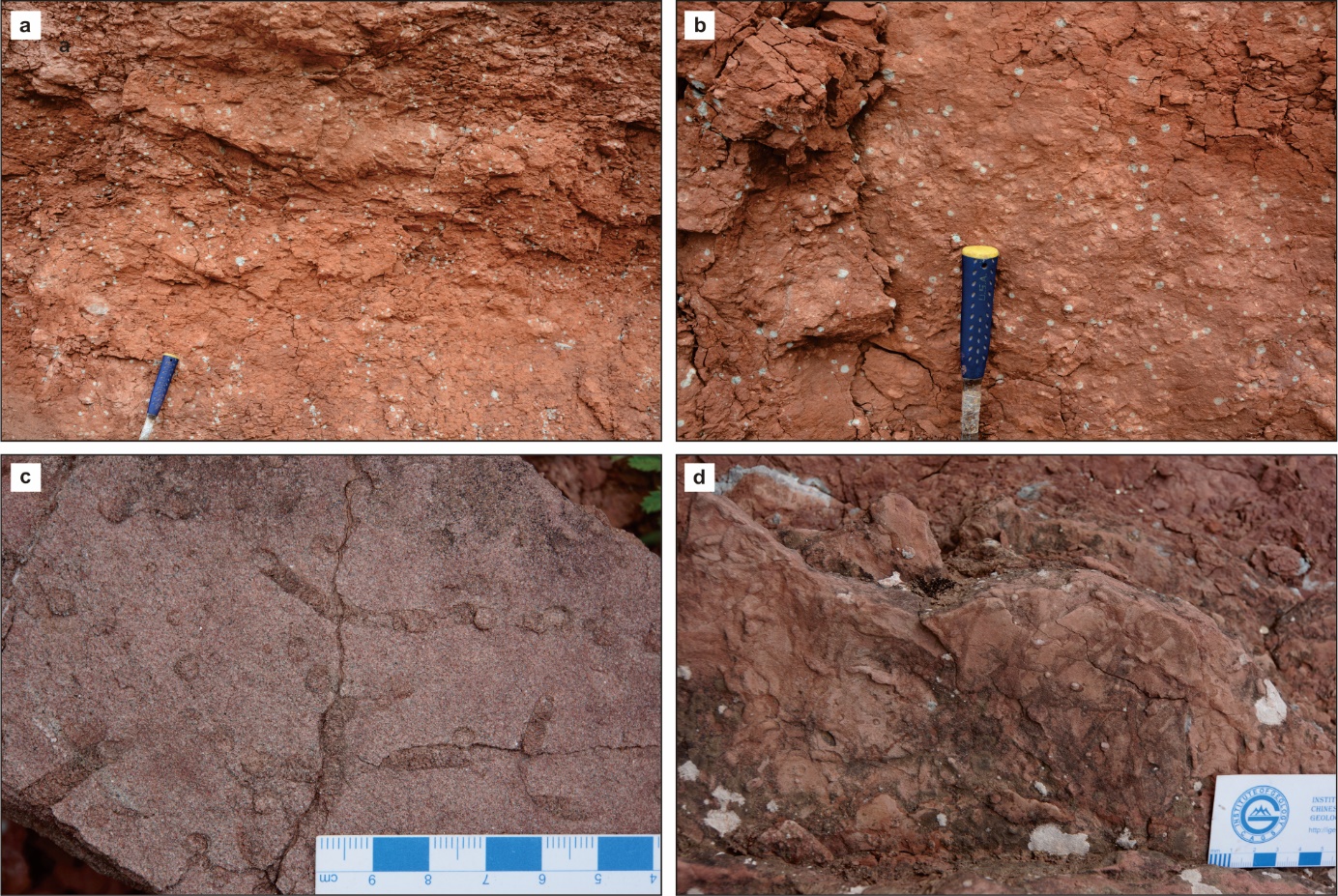


Fig. S2. **U-Pb relative age-probability density diagrams for detrital zircons in the Sunjiagou and Liujiagou formations from northern Shanxi, North China.** Z.C.Z created this figure using CorelDRAW14.

**
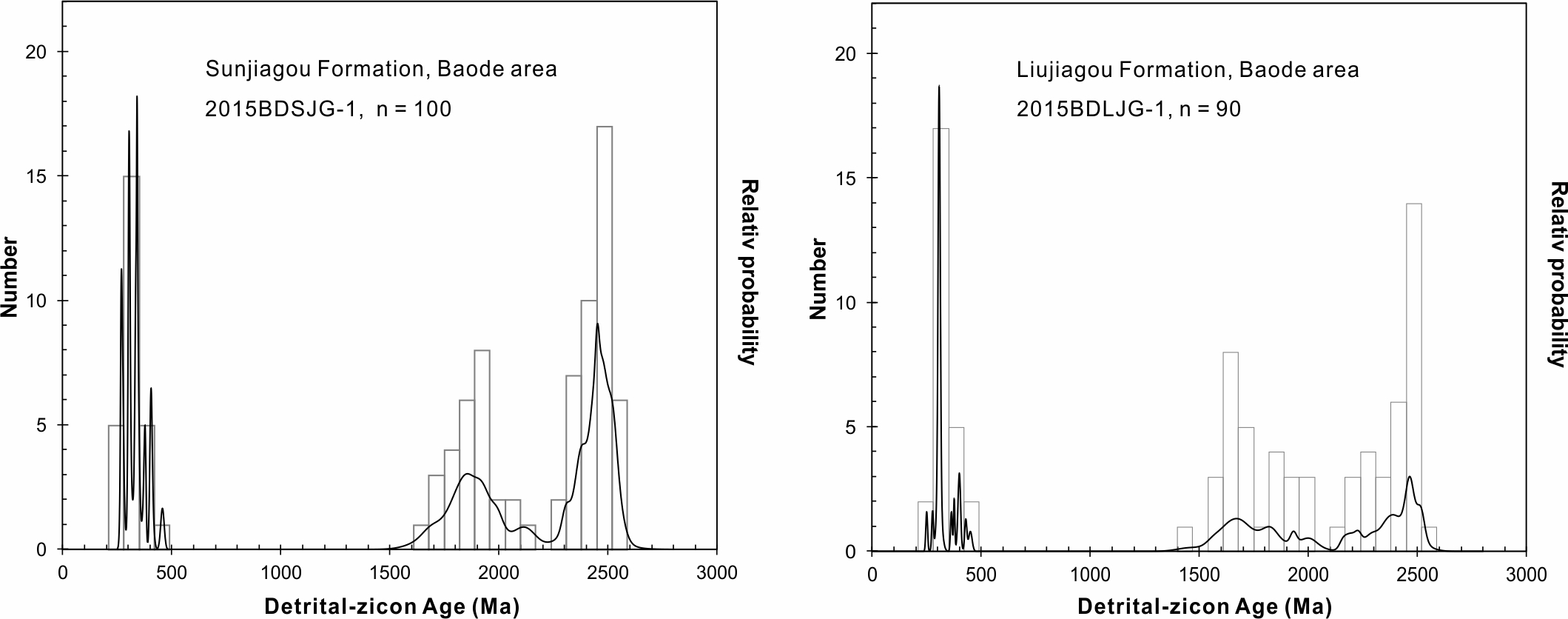
**

Fig. S3. **Carbon and oxygen-isotope cross-plot of samples in the Sunjiagou and Liujiagou formations from northern Shanxi, North China.** Z.C.Z created this figure using CorelDRAW14.


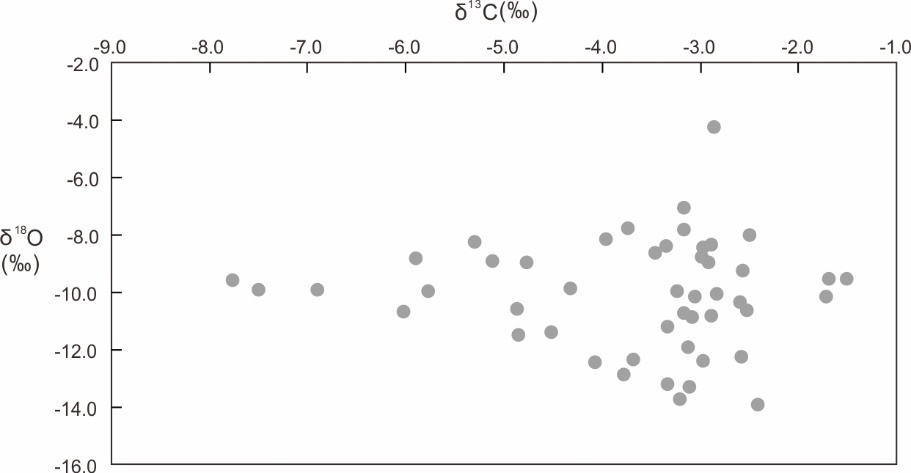


Fig. S4. **Isotopic excursions from typical terrestrial and marine PTB sequences around the world.** (**a**) Lootsberg section in Karoo basin, South Africa^1^. (**b**) Eastern Moscow basin, East Europe^2^. (**c**) Shapeless mountain section in Antarctica^3^. (**d**) Taoshuyuan section in Xinjiang, northwestern China^4^. (**e**) Meishan section (GSSP, Global Stratotype Section and Points) in Zhejiang, South China^4,5^. Z.C.Z created this figure using CorelDRAW14.

**
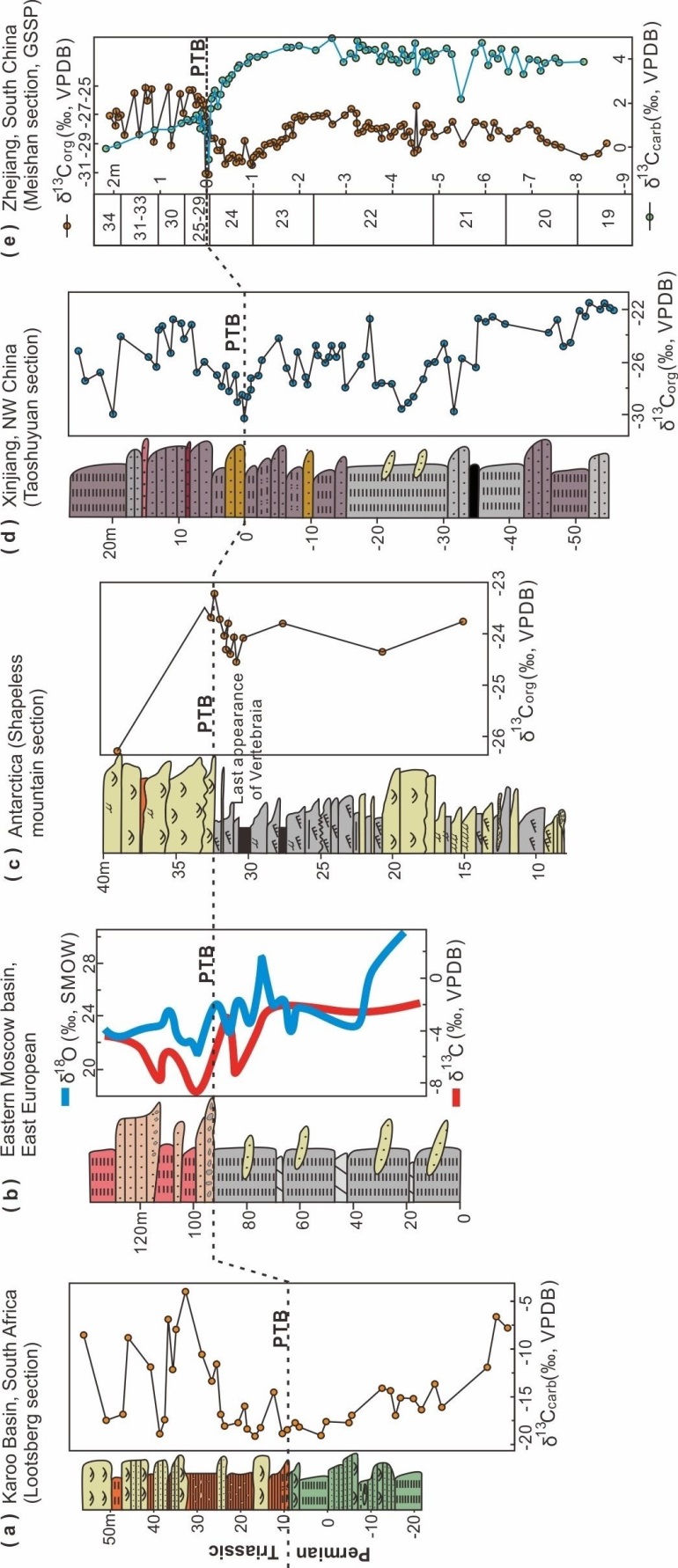
**

Table S1. **Fossils listed in Fig. 2.**

| No. | Fossil | Genus | No. | Fossil | Genus |
| --- | --- | --- | --- | --- | --- |
| 1 | Plant | *Pityospermum* | 46 | Plant | *Danaeopsis-Bernouillia* |
| 2 | Plant | *Algites* | 47 | Plant | *Ctenozamites* |
| 3 | Plant | *Gaussia* | 48 | Conchostracan | *Leptolimnadia* |
| 4 | Plant | *Squarmacarpus* | 49 | Conchostracan | *Paleoleptestheria* |
| 5 | Plant | *Carpolithus* | 50 | Conchostracan | *Loxomegaglypta* |
| 6 | Plant | *Lesleya* | 51 | Conchostracan | *Lioestheria* |
| 7 | Plant | *Esterella* | 52 | Ostracod | *Lutkevichinella* |
| 8 | Plant | *Discinites* | 53 | Ostracod | *Shansinella* |
| 9 | Plant | *Asterophyllites* | 54 | Conchostracan | *Xiangxiella* |
| 10 | Plant | *Carpolithus* | 55 | Conchostracan | *Protomonocarina* |
| 11 | Plant | *Norinia* | 56 | Plant | *Nilssonia* |
| 12 | Plant | *Walchia* | 57 | Conchostracan | *Punctestheria* |
| 13 | Ostracod | *Iniella* | 58 | Conchostracan | *Trisitum* |
| 14 | Ostracod | *Tomiella* | 59 | Conchostracan | *Brachystheria* |
| 15 | Ostracod | *Kemeroviana* | 60 | Conchostracan | *Anyuanestheria* |
| 16 | Conchostracan | *Palaeolimnadiopsis* | 61 | Conchostracan | *Glyptoasmussia* |
| 17 | Conchostracan | *Falsisca* | 62 | Conchostracan | *Dictyostriaca* |
| 18 | Conchostracan | *Huangheetheria* | 63 | Conchostracan | *Loxomegaglypta* |
| 19 | Conchostracan | *Hemicycloleaia* | 64 | Conchostracan | *Leptonemia* |
| 20 | Conchostracan | *Rostroleaia* | 65 | Conchostracan | *Eosolimnadia* |
| 21 | Conchostracan | *Costestheria* | 66 | Plant | *Equisetites* |
| 22 | Conchostracan | *Pemphicyclus* | 67 | Conchostracan | *Triasestheria* |
| 23 | Conchostracan | *Rhyssestheria* | 68 | Conchostracan | *Polygrapta* |
| 24 | Ostracod | *Panxiania* | 69 | Plant | *Lycostrobus-Isoetes* |
| 25 | Plant | *Pseudovoltzia* | 70 | Conchostracan | *Aquilonoglypta* |
| 26 | Plant | *Tatarina* | 71 | Plant | *Schizoneura* |
| 27 | Plant | *Quadrocladus* | 72 | Plant | *Glossotheca-Paleovittaria* |
| 28 | Plant | *Ullmannia* | 73 | Plant | *Otozamites* |
| 29 | Plant | *Sphenophyllum* | 74 | Plant | *Neuropteridium* |
| 30 | Plant | *Callipteris* | 75 | Plant | *Yuccites-"Gangamopteris"* |
| 31 | Plant | *Yuania* | 76 | Plant | *Tongchuanophyllum* |
| 32 | Conchostracan | *Estherina* | 77 | Plant | *Neoglossopteris-"Glossopteris"* |
| 33 | Plant | *Peltaspermum* | 78 | Plant | *Glossophyllum* |
| 34 | Conchostracan | *Sphaerestheria* | 79 | Plant | *Willsiostrobus* |
| 35 | Conchostracan | *Euestheria* | 80 | Plant | *Pleuromeia* |
| 36 | Plant | *Scytophyllum* | 81 | Plant | *Anomopteris-Crematopteris* |
| 37 | Plant | *Pecopteris* | 82 | Plant | *Voltzia* |
| 38 | Plant | *Phyllotheca* | 83 | Conchostracan | *Diaplexa* |
| 39 | Plant | *Taeniopteris* | 84 | Conchostracan | *Liolimnadia* |
| 40 | Conchostracan | *Palaeolimnadia* | 85 | Conchostracan | *Cornia* |
| 41 | Plant | *Calamites* | 86 | Conchostracan | *Gabonestheria* |
| 42 | Plant | *Sphenobaiera* | 87 | Conchostracan | *pseudestheria* |
| 43 | Plant | *Cladophlebis* | 88 | Conchostracan | *Euestheriagutta* |
| 44 | Ostracod | *Darwinula* | 89 | Conchostracan | *Magniestheria* |
| 45 | Ostracod | *Tungchuanina* |  |  |  |

Table S2. **Fossils data from Late Permian to late-Early Triassic in North China.**

| Fossils | Time | Species | Formation-Location | Reference |
| --- | --- | --- | --- | --- |
| Plant | Late Changhsingian | *Ullmannia bronnii* | Lower-middle Upper Sunjiagou Fm-Liulin, Shanxi; Linxian, Shanxi; Yiyang, Henan | ref. 6ref. 6 |
| Plant | Late Changhsingian | *Ullmannia frumentaria* | middle Upper Sunjiagou Fm-Liulin, Shanxi | ref. 6 |
| Plant | Late Changhsingian | *Yuania magnifolia* | Middle Sunjiagou Fm- Liulin, Shanxi | ref. 6 |
| Plant | Late Changhsingian | *Algites junduensis* | middle Upper Sunjiagou Fm-Liulin, Shanxi | ref. 6 |
| Plant | Late Changhsingian | *Sphenophyllum sp.1* | Middle Sunjiagou Fm- Liulin, Shanxi | ref. 6 |
| Plant | Late Changhsingian | *Sphenophyllum sp.2* | middle Upper Sunjiagou Fm-Liulin, Shanxi | ref. 6 |
| Plant | Late Changhsingian | *Sphenophyllum spp.* | Middle Sunjiagou Fm- Liulin, Shanxi | ref. 6 |
| Plant | Late Changhsingian | *Calamites sp.* | middle Upper Sunjiagou Fm-Liulin, Shanxi | ref. 6 |
| Plant | Late Changhsingian | *Asterophyllites equisetofomis* | Middle Sunjiagou Fm- Liulin, Shanxi | ref. 6 |
| Plant | Late Changhsingian | *Phyllotheca sp.* | middle Upper Sunjiagou Fm-Liulin, Shanxi | ref. 6 |
| Plant | Late Changhsingian | *Discinites sunjiagouensis* | Middle Sunjiagou Fm- Liulin, Shanxi | ref. 6 |
| Plant | Late Changhsingian | *Discinites fimbriata* | middle Upper Sunjiagou Fm-Liulin, Shanxi | ref. 6 |
| Plant | Late Changhsingian | *Sphenopteris spp.* | Middle Sunjiagou Fm- Liulin, Shanxi | ref. 6 |
| Plant | Late Changhsingian | *Pecopteris cf. arcuata* | middle Upper Sunjiagou Fm-Liulin, Shanxi | ref. 6 |
| Plant | Late Changhsingian | *Pecopteris spp.* | Middle Sunjiagou Fm- Liulin, Shanxi | ref. 6 |
| Plant | Late Changhsingian | *Cladophlebis sp.* | middle Upper Sunjiagou Fm-Liulin, Shanxi | ref. 6 |
| Plant | Late Changhsingian | *Callipteris lobulata* | Middle Sunjiagou Fm- Liulin, Shanxi | ref. 6 |
| Plant | Late Changhsingian | *Callipteris martinsii* | middle Upper Sunjiagou Fm-Liulin, Shanxi | ref. 6 |
| Plant | Late Changhsingian | *Callipteris papillosa* | Middle Sunjiagou Fm- Liulin, Shanxi | ref. 6 |
| Plant | Late Changhsingian | *Scytophyllum sunjiagouensis* | middle Upper Sunjiagou Fm-Liulin, Shanxi | ref. 6 |
| Plant | Late Changhsingian | *Tatarina cf. sinuosa* | Middle Sunjiagou Fm- Liulin, Shanxi | ref. 6 |
| Plant | Late Changhsingian | *Tatarina mirabilis* | middle Upper Sunjiagou Fm-Liulin, Shanxi | ref. 6 |
| Plant | Late Changhsingian | *Peltaspermum dafengshanensis* | Middle Sunjiagou Fm- Liulin, Shanxi | ref. 6 |
| Plant | Late Changhsingian | *Phylladoderma (Aequistomia) cf. aequalis* | middle Upper Sunjiagou Fm-Liulin, Shanxi | ref. 6 |
| Plant | Late Changhsingian | *Gaussia shanxiensis* | Middle Sunjiagou Fm- Liulin, Shanxi | ref. 6 |
| Plant | Late Changhsingian | *Sphenobaiera micronervis* | middle Upper Sunjiagou Fm-Liulin, Shanxi | ref. 6 |
| Plant | Late Changhsingian | *Walchia sp.* | Middle Sunjiagou Fm- Liulin, Shanxi | ref. 6 |
| Plant | Late Changhsingian | *Pseudovoltzia liebeana* | Middle Sunjiagou Fm- Liulin, Shanxi | ref. 6 |
| Plant | Late Changhsingian | *Quadrocladus solmsii* | Middle Sunjiagou Fm- Liulin, Shanxi | ref. 6 |
| Plant | Late Changhsingian | *Quadrocladus heterodermus* | Middle Sunjiagou Fm- Liulin, Shanxi | ref. 6 |
| Plant | Late Changhsingian | *Quadrocladus sp.* | Middle Sunjiagou Fm- Liulin, Shanxi | ref. 6 |
| Plant | Late Changhsingian | *Pityospermum dafengshanensis* | middle Lower Sunjiagou Fm-Liulin, Shanxi; Linxian,Shanxi; Yiyang, Henan | ref. 6 |
| Plant | Late Changhsingian | *Esterella sp.* | Middle Sunjiagou Fm- Liulin, Shanxi | ref. 6 |
| Plant | Late Changhsingian | *Lesleya anastomosis* | Middle Sunjiagou Fm- Liulin, Shanxi | ref. 6 |
| Plant | Late Changhsingian | *Norinia sp.* | Middle Sunjiagou Fm- Liulin, Shanxi | ref. 6 |
| Plant | Late Changhsingian | *Taeniopteris liulinensis* | Middle Sunjiagou Fm- Liulin, Shanxi | ref. 6 |
| Plant | Late Changhsingian | *Taeniopteris longifolia* | Middle Sunjiagou Fm- Liulin, Shanxi | ref. 6 |
| Plant | Late Changhsingian | *Taeniopteris nystroemii* | Middle Sunjiagou Fm- Liulin, Shanxi | ref. 6 |
| Plant | Late Changhsingian | *Taeniopteris cf. serrulata* | Middle Sunjiagou Fm- Liulin, Shanxi | ref. 6 |
| Plant | Late Changhsingian | *Taeniopteris taiyuanensis* | Middle Sunjiagou Fm- Liulin, Shanxi | ref. 6 |
| Plant | Late Changhsingian | *Cardiocarpus cf.triangularis* | Middle Sunjiagou Fm- Liulin, Shanxi | ref. 6 |
| Plant | Late Changhsingian | *Carpolithus sp.* | Middle Sunjiagou Fm- Liulin, Shanxi | ref. 6 |
| Plant | Late Changhsingian | *Squarmacarpus cuneiformus* | Middle Sunjiagou Fm- Liulin, Shanxi | ref. 6 |
| Plant | Early Spathian | *Pleuromeia rossica* | Upper Liujiagou Fm- Yushe, Shanxi; Jiaocheng, Shanxi | ref. 7 |
| Plant | Early Spathian | *Pleuromeia jiaochengensis* | Upper Liujiagou Fm-Jiaocheng, Shanxi | ref. 7 |
| Plant | Early Spathian | *Neocalamites sp.* | Upper Liujiagou Fm-Jiaocheng, Shanxi | ref. 7 |
| Plant | Early Spathian | *Phyllotheca yusheensis* | Upper Liujiagou Fm- Yushe, Shanxi | ref. 7 |
| Plant | Early Spathian | *Phyllotheca yusheensis* | Upper Liujiagou Fm-Jiaocheng, Shanxi | ref. 7 |
| Plant | Early Spathian | *Crematopteris sp.* | Upper Liujiagou Fm-Jiaocheng, Shanxi | ref. 7 |
| Plant | Early Spathian | *Scyhophyllum sp.* | Upper Liujiagou Fm- Yushe, Shanxi | ref. 7 |
| Plant | Early Spathian | *Yuccites sp.* | Upper Liujiagou Fm- Yushe, Shanxi | ref. 7 |
| Plant | Early Spathian | *Masculostrobus cf. acuminatus* | Upper Liujiagou Fm- Yushe, Shanxi | ref. 7 |
| Plant | Early Spathian | *Neoglossopteris shanxiensis* | Upper Liujiagou Fm- Yushe, Shanxi | ref. 7 |
| Plant | Early Spathian | *Gangamopteris qinshuiensis* | Upper Liujiagou Fm- Yushe, Shanxi | ref. 7 |
| Plant | Early Spathian | *Botrychiopsis (Gondwanidium) sp.* | Upper Liujiagou Fm- Yushe, Shanxi | ref. 7 |
| Plant | Early Spathian | *Palaeovittaria sp.* | Upper Liujiagou Fm- Yushe, Shanxi | ref. 7 |
| Plant | Early Spathian | *Eretmonia sp.* | Upper Liujiagou Fm- Yushe, Shanxi | ref. 7 |
| Plant | Early Spathian | *Samaropsis milleri* | Upper Liujiagou Fm- Yushe, Shanxi | ref. 7 |
| Plant | Middle Spathian | *Pleuromeia epicharis* | Lower Heshanggou Fm- Heshun, Shanxi; Yushe, Shanxi; Yima, Henan | ref. 8 |
| Plant | Middle Spathian-Late Spathian | *Pleuromeia sternbergii* | Heshanggou Fm- Heshun, Shanxi; Yushe, Shanxi; Shouyang, Shanxi; Pingyao, Shanxi; Puxian, Shanxi; Jiyuan, Henan; FengFeng, Hebei; Chengde, Hebei | ref. 8 |
| Plant | Middle Spathian | *Pleuromeia rossica* | Bottom of Heshanggou Fm- Yushe, Shanxi; Jiaocheng, Shanxi | ref. 8 |
| Plant | Middle Spathian | *Isoetites sagittatus* | Lower Heshanggou Fm- Puxian, Shanxi | ref. 8 |
| Plant | Late Spathian | *Annalepis sp.* | Upper Heshanggou Fm- Puxian, Shanxi | ref. 8 |
| Plant | Middle Spathian | *Mesolepidodendron xiabanchengensis* | Lower Heshanggou Fm-Jiyuan, Henan | ref. 8 |
| Plant | Middle Spathian | *Sphenophyllum sp.* | Bottom of Heshanggou Fm- Yushe, Shanxi | ref. 8 |
| Plant | Middle Spathian-Late Spathian | *Calamites shanxiensis* | Heshanggou Fm- Yushe, Shanxi;Shilou, Shanxi;Heshun, Shanxi | ref. 8 |
| Plant | Middle Spathian | *Lobatannufaria sp.* | Bottom of Heshanggou Fm- Yushe, Shanxi | ref. 8 |
| Plant | Middle Spathian-Late Spathian | *Macrostachya gracilis* | Lower-Middle Heshanggou Fm- Heshun, Shanxi | ref. 8 |
| Plant | Middle Spathian | *Phyllotheca bicruris* | Bottom of Heshanggou Fm- Yushe, Shanxi | ref. 8 |
| Plant | Middle Spathian | *Phyllotheca yusheensis* | Lower Heshanggou Fm- Yushe, Shanxi; Shouyang, Shanxi | ref. 8 |
| Plant | Middle Spathian | *Phyllotheca sp.* | Bottom of Heshanggou Fm- Yushe, Shanxi | ref. 8 |
| Plant | Late Spathian | *Neocalamites sp.* | Upper Heshanggou Fm- Puxian, Shanxi | ref. 8 |
| Plant | Middle Spathian | *Equisetites sp.* | Lower Heshanggou Fm- Yushe, Shanxi | ref. 8 |
| Plant | Middle Spathian | *Schizoneura (Eehinostachys) megaphylla* | Lower Heshanggou Fm- Yushe, Shanxi; Heshun, Shanxi | ref. 8 |
| Plant | Middle Spathian | *Anomopteris minima* | Lower Heshanggou Fm- Shouyang, Shanxi | ref. 8 |
| Plant | Late Spathian | *Anomopteris cf. mougeotii* | Upper Heshanggou Fm- Yiyang, Henan | ref. 8 |
| Plant | Middle Spathian | *Crematopteris cf. typic* | Lower Heshanggou Fm- Puxian, Shanxi; Pingyao, Shanxi; Jiyuan, Henan | ref. 8 |
| Plant | Late Spathian | *Crematopteris sp.* | Bottom of Heshanggou Fm- Yushe, Shanxi | ref. 8 |
| Plant | Late Spathian | *Neuropteridium curvinerve* | Bottom of Heshanggou Fm- Yushe, Shanxi | ref. 8 |
| Plant | Middle Spathian-Late Spathian | *Neuropteridium sp.* | Heshanggou Fm- Puxian,Shanxi; Yiyang, Henan | ref. 8 |
| Plant | Middle Spathian | *Ctadophlebis sp.1* | Bottom of Heshanggou Fm- Yushe, Shanxi | ref. 8 |
| Plant | Middle Spathian | *Ctadophlebis sp.2* | Bottom of Heshanggou Fm- Yushe, Shanxi | ref. 8 |
| Plant | Middle Spathian | *sphenopteris yusheensis* | Bottom of Heshanggou Fm- Yushe, Shanxi | ref. 8 |
| Plant | Middle Spathian | *Sphenopterisdelabens* | Bottom of Heshanggou Fm- Yushe, Shanxi | ref. 8 |
| Plant | Middle Spathian-Late Spathian | *Sphenopteris sp.* | Middle Heshanggou Fm- Heshun, Shanxi | ref. 8 |
| Plant | Middle Spathian-Late Spathian | *Glossophylhan sp.* | Heshanggou Fm- Yushe, Shanxi; Yiyang, Henan | ref. 8 |
| Plant | Middle Spathian | *Peltaspermum lobulatum* | Bottom of Heshanggou Fm- Yushe, Shanxi | ref. 8 |
| Plant | Middle Spathian-Late Spathian | *Peltaspermum calycmum* | Lower-Middle Heshanggou Fm- Heshun,Shanxi; Yima, Henan | ref. 8 |
| Plant | Late Spathian | *Scytophyllum cf. bergeri* | Upper Heshanggou Fm- Yiyang, Henan | ref. 8 |
| Plant | Middle Spathian | *Thinnfeldia monopinnata* | Bottom of Heshanggou Fm- Yushe, Shanxi | ref. 8 |
| Plant | Middle Spathian | *Tongchuanophyllum minimum* | Bottom of Heshanggou Fm- Yushe, Shanxi; Shouyang, Shanxi | ref. 8 |
| Plant | Middle Spathian-Late Spathian | *Tongchuanopyllum cf. concinnum* | Lower-Middle Heshanggou Fm- Yushe, Shanxi | ref. 8 |
| Plant | Middle Spathian | *Tongchuanopttum cf. mshensiense* | Lower Heshanggou Fm- Yushe, Shanxi | ref. 8 |
| Plant | Middle Spathian | *Glossopteris shanxiensis* | Bottom of Heshanggou Fm- Yushe, Shanxi | ref. 8 |
| Plant | Middle Spathian-Late Spathian | *Gangamopteris qinshuiensis* | Bottom of Heshanggou Fm- Yushe, Shanxi | ref. 8 |
| Plant | Middle Spathian | *Gangamopteris tuncunensis* | Bottom of Heshanggou Fm- Yushe, Shanxi | ref. 8 |
| Plant | Middle Spathian | *Zamiopteris minor* | Bottom of Heshanggou Fm- Yushe, Shanxi | ref. 8 |
| Plant | Middle Spathian | *Gtossotheca petiolata* | Bottom of Heshanggou Fm- Yushe, Shanxi | ref. 8 |
| Plant | Middle Spathian | *Gtossotheca cuneiformis* | Bottom of Heshanggou Fm- Yushe, Shanxi | ref. 8 |
| Plant | Middle Spathian | *Gtossotheca cochlearis* | Bottom of Heshanggou Fm- Yushe, Shanxi | ref. 8 |
| Plant | Middle Spathian | *Euryphyttum sp.* | Bottom of Heshanggou Fm- Yushe, Shanxi | ref. 8 |
| Plant | Middle Spathian | *Pateovittaria shanxiensis* | Bottom of Heshanggou Fm- Yushe, Shanxi | ref. 8 |
| Plant | Middle Spathian | *Otozamites sp.* | Bottom of Heshanggou Fm- Yushe, Shanxi | ref. 8 |
| Plant | Middle Spathian | *Williamsonia lanceolobata* | Bottom of Heshanggou Fm- Yushe, Shanxi | ref. 8 |
| Plant | Middle Spathian | *Ruehleostachys hongyatouensis* | Lower Heshanggou Fm- Yushe, Shanxi; Heshun, Shanxi | ref. 8 |
| Plant | Middle Spathian | *Voltzia quinquepetala* | Lower Heshanggou Fm- Yushe, Shanxi; Heshun, Shanxi | ref. 8 |
| Plant | Middle Spathian-Late Spathian | *Voltzia cf. heterophylla* | Heshanggou Fm- Yushe,Shanxi;Puxian, Shanxi; Yiyang,Henan | ref. 8 |
| Plant | Middle Spathian | *Willsiostrobus ligulatus* | Lower Heshanggou Fm- Yushe, Shanxi; Heshun, Shanxi | ref. 8 |
| Plant | Middle Spathian-Late Spathian | *Willsiostrobus cordiformis* | middle Lower Heshanggou Fm- Yushe, Shanxi; Heshun, Shanxi | ref. 8 |
| Plant | Middle Spathian | *Willsiostrobus cf. denticulatus* | Lower Heshanggou Fm- Heshun, Shanxi; Puxian, Shanxi | ref. 8 |
| Plant | Middle Spathian | *Yuccites anastomosis* | Lower Heshanggou Fm- Yushe, Shanxi; Heshun, Shanxi; Puxian, Shanxi | ref. 8 |
| Plant | Middle Spathian-Late Spathian | *Tricranolepis obtusiloba* | Bottom of Heshanggou Fm- Yushe, Shanxi | ref. 8 |
| Plant | Middle Spathian | *Tricrananthus sagittatus* | Bottom of Heshanggou Fm- Yushe, Shanxi | ref. 8 |
| Plant | Middle Spathian | *Tricrananthus lobatus* | Lower Heshanggou Fm- Puxian, Shanxi | ref. 8 |
| Plant | Middle Spathian | *Cardiocarpus yuccinoides* | Lower Heshanggou Fm- Yushe,Shanxi; Heshun, Shanxi | ref. 8 |
| Plant | Middle Spathian | *Samaropsis sp.* | Lower Heshanggou Fm- Puxian, Shanxi | ref. 8 |
| Plant | Middle Spathian | *Corticous impression* | Bottom of Heshanggou Fm- Yushe, Shanxi | ref. 8 |
| Ostracode | Latest Changhsingian | *Falsisca eotriassica* | Upper Sunjiagou Fm-Tongchuan,Shaanxi | ref. 9 |
| Ostracode | Latest Changhsingian | *Falsisca postera* | Upper Sunjiagou Fm-Tongchuan,Shaanxi | ref. 9 |
| Ostracode | Latest Changhsingian | *Euestheria gutta* | Upper Sunjiagou Fm-Tongchuan,Shaanxi | ref. 9 |
| Ostracode | Latest Changhsingian | *Euestheria oertlii* | Upper Sunjiagou Fm-Tongchuan,Shaanxi | ref. 9 |
| Ostracode | Latest Changhsingian | *Huangheetheria longellipsa* | Upper Sunjiagou Fm-Tongchuan,Shaanxi | ref. 9 |
| Ostracode | Latest Changhsingian | *Palaeolimnadia sp.* | Upper Sunjiagou Fm-Tongchuan,Shaanxi | ref. 9 |
| Ostracode | Latest Changhsingian | *Hemicycloleaia sunanensis* | Upper Sunan Fm-Sunan, Gansu | ref. 10 |
| Ostracode | Latest Changhsingian | *Hemicycloleaia qinlongensis* | Upper Sunan Fm-Sunan, Gansu | ref. 10 |
| Ostracode | Latest Changhsingian | *Rostroleaia gansuensis* | Upper Sunan Fm-Sunan, Gansu | ref. 10 |
| Ostracode | Latest Changhsingian | *Palaeolimnadia glabra* | Taohaiyingzi Fm-Shaowudameng, Inner Mongolia | ref. 10 |
| Ostracode | Latest Changhsingian | *Palaeolimnadia rossica* | Taohaiyingzi Fm-Shaowudameng, Inner Mongolia | ref. 10 |
| Ostracode | Latest Changhsingian | *Costestheria taohaiyingziensis* | Taohaiyingzi Fm-Shaowudameng, Inner Mongolia | ref. 10 |
| Ostracode | Latest Changhsingian | *Costestheria scoliogabata* | Taohaiyingzi Fm-Shaowudameng, Inner Mongolia | ref. 10 |
| Ostracode | Latest Changhsingian | *Pemphicyclus baiyintalaensis* | Taohaiyingzi Fm-Shaowudameng, Inner Mongolia | ref. 10 |
| Ostracode | Latest Changhsingian | *Penphicyclus trochoides* | Taohaiyingzi Fm-Shaowudameng, Inner Mongolia | ref. 10 |
| Ostracode | Latest Changhsingian | *Penphicyclus cf.arangastachus* | Taohaiyingzi Fm-Shaowudameng,Inner Mongolia | ref. 10 |
| Ostracode | Latest Changhsingian | *Palaeolimnadiopsis deminuta* | Taohaiyingzi Fm-Shaowudameng, Inner Mongolia | ref. 10 |
| Ostracode | Latest Changhsingian | *Rhyssestheria lampra* | Taohaiyingzi Fm-Shaowudameng, Inner Mongolia | ref. 10 |
| Ostracode | Latest Changhsingian | *Rhyssestheria perfecta* | Taohaiyingzi Fm-Shaowudameng, Inner Mongolia | ref. 10 |
| Ostracode | Latest Changhsingian | *Sphaerestheria cf. sibirica* | Taohaiyingzi Fm-Shaowudameng, Inner Mongolia | ref. 10 |
| Ostracode | Latest Changhsingian | *Estherina aspred* | Taohaiyingzi Fm-Shaowudameng, Inner Mongolia | ref. 10 |
| Ostracode | Latest Changhsingian | *Leptolimnadia shanxiensis* | Upper Liujiagou Fm-Jiaocheng, Shanxi | ref. 11 |
| Conchostracan | Latest Changhsingian | *Falsisca eotriassica* | Upper Sunjiagou Fm-Tongchuan, Shaanxi | ref. 9 |
| Conchostracan | Latest Changhsingian | *Falsisca postera* | Upper Sunjiagou Fm-Tongchuan, Shaanxi | ref. 9 |
| Conchostracan | Latest Changhsingian | *Euestheria gutta* | Upper Sunjiagou Fm-Tongchuan, Shaanxi | ref. 9 |
| Conchostracan | Latest Changhsingian | *Euestheria oertlii* | Upper Sunjiagou Fm-Tongchuan, Shaanxi | ref. 9 |
| Conchostracan | Latest Changhsingian | *Huangheetheria longellipsa* | Upper Sunjiagou Fm-Tongchuan, Shaanxi | ref. 9 |
| Conchostracan | Latest Changhsingian | *Palaeolimnadia sp.* | Upper Sunjiagou Fm-Tongchuan, Shaanxi | ref. 9 |
| Conchostracan | Latest Changhsingian-Griesbachian | *pseudestheria minuta* | Upper Sunjiagou Fm-Shhuanhe, Liulin, Shanxi | ref. 12 |
| Conchostracan | Latest Changhsingian-Griesbachian | *pseudestheria xinjiangensis* | Upper Sunjiagou Fm-Shhuanhe, Liulin, Shanxi | ref. 12 |
| Conchostracan | Latest Changhsingian-Griesbachian | *Euestheriagutta* | Upper Sunjiagou Fm-Shhuanhe, Liulin, Shanxi | ref. 12 |
| Conchostracan | Latest Changhsingian-Griesbachian | *E. nordvikensis* | Upper Sunjiagou Fm-Shhuanhe, Liulin, Shanxi | ref. 12 |
| Conchostracan | Latest Changhsingian-Griesbachian | *E. sp.* | Upper Sunjiagou Fm-Shhuanhe, Liulin, Shanxi | ref. 12 |
| Conchostracan | Latest Changhsingian-Griesbachian | *Palaeolimnadi-opsis vilujensis* | Upper Sunjiagou Fm-Shhuanhe, Liulin, Shanxi | ref. 12 |
| Conchostracan | Latest Changhsingian-Griesbachian | *P.sp* | Upper Sunjiagou Fm-Liulin, Shanxi | ref. 12 |
| Conchostracan | Latest Changhsingian-Griesbachian | *Magniestheria mangaliensis* | Upper Sunjiagou Fm-Liulin, Shanxi | ref. 12 |
| Conchostracan | Latest Changhsingian-Griesbachian | *Magniestheria subcircularis* | Upper Sunjiagou Fm-Liulin, Shanxi | ref. 12 |
| Conchostracan | Latest Changhsingian-Griesbachian | *M. sp. 2* | Upper Sunjiagou Fm-Liulin, Shanxi | ref. 12 |
| Conchostracan | Latest Changhsingian | *Hemicycloleaia sunanensis* | Upper Sunan Fm-Sunan, Gansu | ref. 13 |
| Conchostracan | Latest Changhsingian | *Hemicycloleaia qinlongensis* | Upper Sunan Fm-Sunan, Gansu | ref. 13 |
| Conchostracan | Latest Changhsingian | *Rostroleaia gansuensis* | Upper Sunan Fm-Sunan, Gansu | ref. 13 |
| Conchostracan | Late Changhsingian | *Palaeolimnadia glabra* | Taohaiyingzi Fm-Shaowudameng, Inner Mongolia | ref. 10 |
| Conchostracan | Late Changhsingian | *Palaeolimnadia rossica* | Taohaiyingzi Fm-Shaowudameng, Inner Mongolia | ref. 10 |
| Conchostracan | Late Changhsingian | *Costestheria taohaiyingziensis* | Taohaiyingzi Fm-Shaowudameng, Inner Mongolia | ref. 10 |
| Conchostracan | Late Changhsingian | *Costestheria scoliogabata* | Taohaiyingzi Fm-Shaowudameng, Inner Mongolia | ref. 10 |
| Conchostracan | Late Changhsingian | *Pemphicyclus baiyintalaensis* | Taohaiyingzi Fm-Shaowudameng, Inner Mongolia | ref. 10 |
| Conchostracan | Late Changhsingian | *Penphicyclus trochoides* | Taohaiyingzi Fm-Shaowudameng, Inner Mongolia | ref. 10 |
| Conchostracan | Late Changhsingian | *Penphicyclus cf. arangastachus* | Taohaiyingzi Fm-Shaowudameng, Inner Mongolia | ref. 10 |
| Conchostracan | Late Changhsingian | *Palaeolimnadiopsis deminuta* | Taohaiyingzi Fm-Shaowudameng, Inner Mongolia | ref. 10 |
| Conchostracan | Late Changhsingian | *Rhyssestheria lampra* | Taohaiyingzi Fm-Shaowudameng, Inner Mongolia | ref. 10 |
| Conchostracan | Late Changhsingian | *Rhyssestheria perfecta* | Taohaiyingzi Fm-Shaowudameng, Inner Mongolia | ref. 10 |
| Conchostracan | Late Changhsingian | *Sphaerestheria cf. sibirica* | Taohaiyingzi Fm-Shaowudameng, Inner Mongolia | ref. 10 |
| Conchostracan | Late Changhsingian | *Estherina aspred* | Taohaiyingzi Fm-Shaowudameng, Inner Mongolia | ref. 10 |
| Conchostracan | Early Spathian | *Leptolimnadia shanxiensis* | Upper Liujiagou Fm-Jiaocheng, Shanxi | ref. 11 |
| Conchostracan | Early Spathian | *Leptolimnadia jiaochengensis* | Upper Liujiagou Fm-Jiaocheng, Shanxi | ref. 11 |
| Conchostracan | Early Spathian | *Palaeolimnadia komiana* | Upper Liujiagou Fm-Jiaocheng, Shanxi | ref. 11 |
| Conchostracan | Early Spathian | *Palaeolimnadia chuanbeiensis* | Upper Liujiagou Fm-Jiaocheng, Shanxi | ref. 11 |
| Conchostracan | Early Spathian | *Palaeolimnadia multilineata* | Upper Liujiagou Fm-Jiaocheng, Shanxi | ref. 11 |
| Conchostracan | Early Spathian | *Palaeolimnadiacontracta* | Upper Liujiagou Fm-Jiaocheng,Shanxi | ref. 11 |
| Conchostracan | Early Spathian | *Lioestheria jiaochengensis* | Upper Liujiagou Fm-Jiaocheng, Shanxi | ref. 11 |
| Conchostracan | Early Spathian | *Paleoleptestheria endybalica* | Upper Liujiagou Fm-Jiaocheng, Shanxi | ref. 11 |
| Conchostracan | Early Spathian | *Loxomegaglypta jiaochengensis* | Upper Liujiagou Fm-Jiaocheng, Shanxi | ref. 11 |
| Conchostracan | Late Spathian | *Glyptoasmussia quadrata* | Upper Heshanggou Fm-Hancheng, Shaanxi | ref. 8 |
| Conchostracan | Late Spathian | *Polygrapta subelliptica* | Upper Heshanggou Fm-Hancheng, Shaanxi | ref. 8 |
| Conchostracan | Late Spathian | *Polygrapta xuefengchuanensis* | Upper Heshanggou Fm-Hancheng, Shaanxi | ref. 8 |
| Conchostracan | Middle Spathian- Late Spathian | *Palaeolimnadia ovata* | Middle-Upper Heshanggou Fm-Hancheng, Shaanxi | ref. 8 |
| Conchostracan | Middle Spathian- Late Spathian | *Palaeolimnadia longovata* | Upper Heshanggou Fm-Hancheng, Shaanxi | ref. 8 |
| Conchostracan | Middle Spathian- Late Spathian | *Liolimnadia jiamijiawanensis* | Middle-Upper Heshanggou Fm-Hancheng, Shaanxi | ref. 8 |
| Conchostracan | Middle Spathian- Late Spathian | *Cornia guchengensis* | Middle-Upper Heshanggou Fm-Hancheng, Shaanxi | ref. 8 |
| Conchostracan | Middle Spathian- Late Spathian | *Gabonestheria clinotuberica* | Middle-Upper Heshanggou Fm-Hancheng, Shaanxi | ref. 8 |
| Conchostracan | Middle Spathian- Late Spathian | *Gabonestheria guchengchuanensis* | Middle-Upper Heshanggou Fm-Hancheng, Shaanxi | ref. 8 |
| Conchostracan | Late Spathian | *Aquilonoglypta clinoquadrata* | Upper Heshanggou Fm-Hancheng, Shaanxi | ref. 8 |
| Conchostracan | Late Spathian | *Aquilonoglypta xilougouensis* | Upper Heshanggou Fm-Hancheng, Shaanxi | ref. 8 |
| Conchostracan | Middle Spathian- Late Spathian | *Dictyostriaca subcyclata* | Middle-Upper Heshanggou Fm-Hancheng, Shaanxi | ref. 8 |
| Conchostracan | Late Spathian | *Loxomegaglypta sangerjiagouensis* | Upper Heshanggou Fm-Hancheng, Shaanxi | ref. 8 |
| Conchostracan | Middle Spathian- Late Spathian | *Leptonemia cyclata* | Middle-Upper Heshanggou Fm-Hancheng, Shaanxi | ref. 8 |
| Conchostracan | Middle Spathian- Late Spathian | *Diaplexa varidicta* | Middle-Upper Heshanggou Fm-Hancheng, Shaanxi | ref. 8 |
| Conchostracan | Late Spathian | *Lioestheria hanchengensis* | Upper Heshanggou Fm-Hancheng, Shaanxi | ref. 8 |
| Conchostracan | Late Spathian | *Palaeolimnadia magnapicalis* | Upper Heshanggou Fm-Hancheng, Shaanxi | ref. 14 |
| Conchostracan | Late Spathian | *Eosolimnadia subquadrata* | Upper Heshanggou Fm-Hancheng, Shaanxi | ref. 14 |
| Conchostracan | Late Spathian | *Eosolimnadia xingxianensis* | Upper Heshanggou Fm-Hancheng, Shaanxi | ref. 14 |
| Conchostracan | Late Spathian | *Eosolimnadia shanxiensis* | Upper Heshanggou Fm-Hancheng, Shaanxi | ref. 14 |
| Conchostracan | Late Spathian | *Triasestheria shanxiensis* | Upper Heshanggou Fm-Hancheng, Shaanxi | ref. 14 |

Table S3. **The volume size of *Darwinula* specimens from Late Changhsingian to Spathian.**

| No. | Length | Hight | Thickness | Volume | Time | Data source |
| --- | --- | --- | --- | --- | --- | --- |
| 1 | 760 | 410 | 375 | 7.786407114 | Late Changhsingian-1 | ref. 9 |
| 2 | 618 | 380 | 365 | 7.651843334 | Late Changhsingian-1 | ref. 9 |
| 3 | 704 | 384 | 350 | 7.694750326 | Late Changhsingian-1 | ref. 9 |
| 4 | 733 | 400 | 360 | 7.742244864 | Late Changhsingian-1 | ref. 9 |
| 5 | 750 | 300 | 275 | 7.51029361 | Late Changhsingian-1 | ref. 9 |
| 6 | 630 | 390 | 355 | 7.659411907 | Late Changhsingian-1 | ref. 9 |
| 7 | 713 | 278 | 220 | 7.358335404 | Late Changhsingian-1 | ref. 9 |
| 8 | 600 | 280 | 245 | 7.333253764 | Late Changhsingian-1 | ref. 9 |
| 9 | 742 | 400 | 355 | 7.741470647 | Late Changhsingian-1 | ref. 9 |
| 10 | 723 | 410 | 375 | 7.764731819 | Late Changhsingian-1 | ref. 9 |
| 11 | 765 | 400 | 355 | 7.754728177 | Late Changhsingian-1 | ref. 9 |
| 12 | 630 | 310 | 265 | 7.432726515 | Late Changhsingian-1 | ref. 9 |
| 13 | 600 | 300 | 310 | 7.465412597 | Late Changhsingian-1 | ref. 9 |
| 14 | 601 | 300 | 260 | 7.389747472 | Late Changhsingian-1 | ref. 9 |
| 15 | 661 | 380 | 355 | 7.668991807 | Late Changhsingian-1 | ref. 9 |
| 16 | 630 | 305 | 270 | 7.433782551 | Late Changhsingian-1 | ref. 9 |
| 17 | 545 | 298 | 255 | 7.335931344 | Late Changhsingian-1 | ref. 9 |
| 18 | 690 | 310 | 300 | 7.526110437 | Late Changhsingian-1 | ref. 9 |
| 19 | 791 | 400 | 331 | 7.738842866 | Late Changhsingian-1 | ref. 9 |
| 20 | 672 | 385 | 355 | 7.681836753 | Late Changhsingian-1 | ref. 9 |
| 21 | 700 | 365 | 350 | 7.670237347 | Late Changhsingian-1 | ref. 9 |
| 22 | 765 | 350 | 313 | 7.642052215 | Late Changhsingian-1 | ref. 9 |
| 23 | 720 | 320 | 300 | 7.558382127 | Late Changhsingian-1 | ref. 9 |
| 24 | 695 | 385 | 365 | 7.708516796 | Late Changhsingian-1 | ref. 9 |
| 25 | 660 | 350 | 300 | 7.559511632 | Late Changhsingian-1 | ref. 9 |
| 26 | 705 | 325 | 305 | 7.563150715 | Late Changhsingian-1 | ref. 9 |
| 27 | 666 | 380 | 355 | 7.672264577 | Late Changhsingian-1 | ref. 9 |
| 28 | 730 | 343 | 313 | 7.612939715 | Late Changhsingian-1 | ref. 9 |
| 29 | 585 | 298 | 250 | 7.358090537 | Late Changhsingian-1 | ref. 9 |
| 30 | 655 | 327 | 285 | 7.50441231 | Late Changhsingian-1 | ref. 9 |
| 31 | 667 | 267 | 230 | 7.331143329 | Late Changhsingian-1 | ref. 9 |
| 32 | 407 | 200 | 170 | 6.859851724 | Late Changhsingian-1 | ref. 9 |
| 33 | 660 | 270 | 220 | 7.312108778 | Late Changhsingian-1 | ref. 9 |
| 34 | 555 | 210 | 175 | 7.028328724 | Late Changhsingian-1 | ref. 9 |
| 35 | 771 | 310 | 270 | 7.528558234 | Late Changhsingian-1 | ref. 9 |
| 36 | 810 | 352 | 310 | 7.665167774 | Late Changhsingian-1 | ref. 9 |
| 37 | 600 | 285 | 255 | 7.358314689 | Late Changhsingian-1 | ref. 9 |
| 38 | 630 | 310 | 280 | 7.456638672 | Late Changhsingian-1 | ref. 9 |
| 39 | 690 | 300 | 275 | 7.474081437 | Late Changhsingian-1 | ref. 9 |
| 40 | 780 | 330 | 295 | 7.599208956 | Late Changhsingian-1 | ref. 9 |
| 41 | 630 | 300 | 265 | 7.418486076 | Late Changhsingian-1 | ref. 9 |
| 42 | 600 | 310 | 280 | 7.435449373 | Late Changhsingian-1 | ref. 9 |
| 43 | 700 | 350 | 320 | 7.61309446 | Late Changhsingian-1 | ref. 9 |
| 44 | 630 | 300 | 275 | 7.434572896 | Late Changhsingian-1 | ref. 9 |
| 45 | 600 | 250 | 225 | 7.247052175 | Late Changhsingian-1 | ref. 9 |
| 46 | 700 | 300 | 275 | 7.480330386 | Late Changhsingian-1 | ref. 9 |
| 47 | 700 | 300 | 275 | 7.480330386 | Late Changhsingian-1 | ref. 9 |
| 48 | 800 | 400 | 375 | 7.797959644 | Late Changhsingian-1 | ref. 9 |
| 49 | 600 | 250 | 225 | 7.247052175 | Late Changhsingian-1 | ref. 9 |
| 50 | 620 | 380 | 350 | 7.635021728 | Late Changhsingian-1 | ref. 9 |
| 51 | 650 | 330 | 305 | 7.534505534 | Late Changhsingian-1 | ref. 9 |
| 52 | 600 | 280 | 250 | 7.342027688 | Late Changhsingian-1 | ref. 9 |
| 53 | 750 | 400 | 385 | 7.781360382 | Late Changhsingian-1 | ref. 9 |
| 54 | 650 | 250 | 225 | 7.281814281 | Late Changhsingian-1 | ref. 9 |
| 55 | 825 | 400 | 375 | 7.811323605 | Late Changhsingian-1 | ref. 9 |
| 56 | 600 | 300 | 275 | 7.413383597 | Late Changhsingian-1 | ref. 9 |
| 57 | 700 | 350 | 320 | 7.61309446 | Late Changhsingian-1 | ref. 9 |
| 58 | 550 | 275 | 250 | 7.29641379 | Late Changhsingian-1 | ref. 9 |
| 59 | 775 | 375 | 350 | 7.726179412 | Late Changhsingian-1 | ref. 9 |
| 60 | 700 | 400 | 380 | 7.745720026 | Late Changhsingian-1 | ref. 9 |
| 61 | 650 | 350 | 325 | 7.58764316 | Late Changhsingian-1 | ref. 9 |
| 62 | 625 | 300 | 275 | 7.431112364 | Late Changhsingian-1 | ref. 9 |
| 63 | 650 | 300 | 275 | 7.448145703 | Late Changhsingian-1 | ref. 9 |
| 64 | 725 | 325 | 300 | 7.56812102 | Late Changhsingian-1 | ref. 9 |
| 65 | 800 | 300 | 275 | 7.538322333 | Late Changhsingian-1 | ref. 9 |
| 66 | 550 | 250 | 225 | 7.209263614 | Late Changhsingian-1 | ref. 9 |
| 67 | 600 | 250 | 230 | 7.256597493 | Late Changhsingian-1 | ref. 9 |
| 68 | 550 | 250 | 225 | 7.209263614 | Late Changhsingian-1 | ref. 9 |
| 69 | 647 | 290 | 253 | 7.395201197 | Late Changhsingian-1 | ref. 9 |
| 70 | 700 | 360 | 345 | 7.657998034 | Late Changhsingian-1 | ref. 9 |
| 71 | 605 | 305 | 285 | 7.439678472 | Late Changhsingian-1 | ref. 9 |
| 72 | 654 | 380 | 365 | 7.676432607 | Late Changhsingian-1 | ref. 9 |
| 73 | 770 | 410 | 385 | 7.803513709 | Late Changhsingian-1 | ref. 9 |
| 74 | 690 | 390 | 365 | 7.71098496 | Late Changhsingian-1 | ref. 9 |
| 75 | 685 | 325 | 295 | 7.536174346 | Late Changhsingian-1 | ref. 9 |
| 76 | 654 | 325 | 300 | 7.523360762 | Late Changhsingian-1 | ref. 9 |
| 77 | 720 | 360 | 335 | 7.657458202 | Late Changhsingian-1 | ref. 9 |
| 78 | 577 | 311 | 290 | 7.435112598 | Late Changhsingian-1 | ref. 9 |
| 79 | 685 | 371 | 350 | 7.667910923 | Late Changhsingian-1 | ref. 9 |
| 80 | 630 | 381 | 355 | 7.649272276 | Late Changhsingian-1 | ref. 9 |
| 81 | 670 | 350 | 320 | 7.594071223 | Late Changhsingian-1 | ref. 9 |
| 82 | 630 | 360 | 335 | 7.599466255 | Late Changhsingian-1 | ref. 9 |
| 83 | 650 | 450 | 435 | 7.823393525 | Late Changhsingian-1 | ref. 9 |
| 84 | 900 | 300 | 275 | 7.589474856 | Late Changhsingian-1 | ref. 9 |
| 85 | 500 | 275 | 250 | 7.255021105 | Late Changhsingian-1 | ref. 9 |
| 86 | 600 | 257 | 214 | 7.238097332 | Late Changhsingian-1 | ref. 9 |
| 87 | 655 | 245 | 205 | 7.235479121 | Late Changhsingian-1 | ref. 9 |
| 88 | 683 | 329 | 280 | 7.518616468 | Late Changhsingian-1 | ref. 9 |
| 89 | 672 | 264 | 216 | 7.302205349 | Late Changhsingian-1 | ref. 9 |
| 90 | 625 | 287.5 | 237.5 | 7.348959878 | Late Changhsingian-1 | ref. 9 |
| 91 | 575 | 300 | 262.5 | 7.374696805 | Late Changhsingian-1 | ref. 9 |
| 92 | 600 | 275 | 200 | 7.237292338 | Late Changhsingian-1 | ref. 9 |
| 93 | 425 | 212.5 | 175 | 6.917564311 | Late Changhsingian-1 | ref. 9 |
| 94 | 343 | 171 | 157 | 6.684269451 | Late Changhsingian-1 | ref. 9 |
| 95 | 275 | 175 | 163 | 6.612002506 | Late Changhsingian-1 | ref. 9 |
| 96 | 525 | 200 | 188 | 7.012968969 | Late Changhsingian-1 | ref. 9 |
| 97 | 500 | 250 | 220 | 7.158111092 | Late Changhsingian-1 | ref. 9 |
| 98 | 417 | 200 | 175 | 6.8826352 | Late Changhsingian-1 | ref. 9 |
| 99 | 390 | 181 | 162 | 6.77719688 | Late Changhsingian-1 | ref. 9 |
| 100 | 660 | 330 | 300 | 7.533957528 | Late Changhsingian-1 | ref. 9 |
| 101 | 650 | 300 | 300 | 7.485934264 | Late Changhsingian-1 | ref. 9 |
| 102 | 600 | 300 | 270 | 7.405414667 | Late Changhsingian-1 | ref. 9 |
| 103 | 500 | 275 | 235 | 7.228148958 | Late Changhsingian-1 | ref. 9 |
| 104 | 650 | 300 | 265 | 7.432058883 | Late Changhsingian-1 | ref. 9 |
| 105 | 600 | 250 | 225 | 7.247052175 | Late Changhsingian-1 | ref. 9 |
| 106 | 600 | 300 | 300 | 7.451172158 | Late Changhsingian-1 | ref. 9 |
| 107 | 800 | 250 | 250 | 7.417748402 | Late Changhsingian-1 | ref. 9 |
| 108 | 650 | 350 | 300 | 7.552881053 | Late Changhsingian-1 | ref. 9 |
| 109 | 500 | 300 | 250 | 7.292809665 | Late Changhsingian-1 | ref. 9 |
| 110 | 500 | 250 | 250 | 7.213628419 | Late Changhsingian-1 | ref. 9 |
| 111 | 500 | 200 | 200 | 7.019808393 | Late Changhsingian-2 | ref. 9 |
| 112 | 700 | 300 | 270 | 7.472361457 | Late Changhsingian-2 | ref. 9 |
| 113 | 450 | 300 | 260 | 7.264085514 | Late Changhsingian-2 | ref. 9 |
| 114 | 600 | 300 | 250 | 7.371990911 | Late Changhsingian-2 | ref. 9 |
| 115 | 700 | 250 | 250 | 7.359756455 | Late Changhsingian-2 | ref. 9 |
| 116 | 700 | 240 | 240 | 7.324298921 | Late Changhsingian-2 | ref. 9 |
| 117 | 650 | 250 | 230 | 7.291359599 | Late Changhsingian-2 | ref. 9 |
| 118 | 550 | 250 | 230 | 7.218808932 | Late Changhsingian-2 | ref. 9 |
| 119 | 600 | 350 | 300 | 7.518118947 | Late Changhsingian-2 | ref. 9 |
| 120 | 750 | 400 | 375 | 7.76993092 | Late Changhsingian-2 | ref. 9 |
| 121 | 700 | 300 | 275 | 7.480330386 | Late Changhsingian-2 | ref. 9 |
| 122 | 650 | 400 | 375 | 7.707783013 | Late Changhsingian-2 | ref. 9 |
| 123 | 650 | 280 | 265 | 7.40209566 | Late Changhsingian-2 | ref. 9 |
| 124 | 450 | 225 | 200 | 7.025203425 | Late Changhsingian-2 | ref. 9 |
| 125 | 850 | 450 | 420 | 7.924659128 | Late Changhsingian-2 | ref. 9 |
| 126 | 750 | 350 | 325 | 7.649791066 | Late Changhsingian-2 | ref. 9 |
| 127 | 650 | 300 | 275 | 7.448145703 | Late Changhsingian-2 | ref. 9 |
| 128 | 700 | 350 | 325 | 7.619827843 | Late Changhsingian-2 | ref. 9 |
| 129 | 550 | 280 | 265 | 7.329544992 | Late Changhsingian-2 | ref. 9 |
| 130 | 600 | 300 | 275 | 7.413383597 | Late Changhsingian-2 | ref. 9 |
| 131 | 650 | 280 | 265 | 7.40209566 | Late Changhsingian-2 | ref. 9 |
| 132 | 700 | 290 | 265 | 7.44952031 | Late Changhsingian-2 | ref. 9 |
| 133 | 600 | 300 | 275 | 7.413383597 | Late Changhsingian-2 | ref. 9 |
| 134 | 720 | 350 | 325 | 7.632062299 | Late Changhsingian-2 | ref. 9 |
| 135 | 600 | 250 | 225 | 7.247052175 | Late Changhsingian-2 | ref. 9 |
| 136 | 650 | 300 | 275 | 7.448145703 | Late Changhsingian-2 | ref. 9 |
| 137 | 600 | 250 | 225 | 7.247052175 | Late Changhsingian-2 | ref. 9 |
| 138 | 650 | 300 | 275 | 7.448145703 | Late Changhsingian-2 | ref. 9 |
| 139 | 840 | 360 | 330 | 7.717874124 | Late Changhsingian-2 | ref. 9 |
| 140 | 600 | 300 | 270 | 7.405414667 | Late Changhsingian-2 | ref. 9 |
| 141 | 443 | 261 | 235 | 7.152740072 | Late Changhsingian-2 | ref. 9 |
| 142 | 625 | 225 | 200 | 7.167870929 | Late Changhsingian-2 | ref. 9 |
| 143 | 570 | 270 | 240 | 7.286228259 | Late Changhsingian-2 | ref. 9 |
| 144 | 720 | 270 | 150 | 7.183565917 | Late Changhsingian-2 | ref. 9 |
| 145 | 720 | 375 | 330 | 7.668656102 | Late Changhsingian-2 | ref. 9 |
| 146 | 660 | 285 | 240 | 7.373378435 | Late Changhsingian-2 | ref. 9 |
| 147 | 660 | 300 | 255 | 7.421983768 | Late Changhsingian-2 | ref. 9 |
| 148 | 660 | 315 | 270 | 7.467996651 | Late Changhsingian-2 | ref. 9 |
| 149 | 600 | 270 | 240 | 7.308504654 | Late Changhsingian-2 | ref. 9 |
| 150 | 320 | 160 | 136 | 6.561587267 | Late Changhsingian-2 | ref. 9 |
| 151 | 615 | 240 | 195 | 7.177899367 | Late Changhsingian-2 | ref. 9 |
| 152 | 630 | 270 | 240 | 7.329693953 | Late Changhsingian-2 | ref. 9 |
| 153 | 720 | 300 | 270 | 7.484595913 | Late Changhsingian-2 | ref. 9 |
| 154 | 660 | 270 | 240 | 7.349897339 | Late Changhsingian-2 | ref. 9 |
| 155 | 600 | 270 | 240 | 7.308504654 | Late Changhsingian-2 | ref. 9 |
| 156 | 630 | 210 | 195 | 7.130372853 | Late Changhsingian-2 | ref. 9 |
| 157 | 495 | 210 | 180 | 6.990875396 | Late Changhsingian-2 | ref. 9 |
| 158 | 667 | 300 | 267 | 7.445777126 | Late Changhsingian-2 | ref. 9 |
| 159 | 700 | 300 | 283 | 7.493295363 | Late Changhsingian-2 | ref. 9 |
| 160 | 650 | 300 | 300 | 7.485934264 | Late Changhsingian-2 | ref. 9 |
| 161 | 600 | 275 | 275 | 7.375595036 | Late Changhsingian-2 | ref. 9 |
| 162 | 650 | 350 | 350 | 7.619827843 | Late Changhsingian-2 | ref. 9 |
| 163 | 800 | 400 | 365 | 7.78622124 | Late Changhsingian-2 | ref. 9 |
| 164 | 750 | 250 | 225 | 7.343962188 | Late Changhsingian-2 | ref. 9 |
| 165 | 600 | 270 | 250 | 7.326233421 | Late Changhsingian-2 | ref. 9 |
| 166 | 700 | 300 | 285 | 7.495842552 | Late Changhsingian-2 | ref. 9 |
| 167 | 500 | 200 | 185 | 6.985950126 | Late Changhsingian-2 | ref. 9 |
| 168 | 700 | 280 | 265 | 7.434280343 | Late Changhsingian-2 | ref. 9 |
| 169 | 720 | 320 | 305 | 7.565560712 | Late Changhsingian-2 | ref. 9 |
| 170 | 600 | 300 | 285 | 7.428895763 | Late Changhsingian-2 | ref. 9 |
| 171 | 650 | 280 | 265 | 7.40209566 | Late Changhsingian-2 | ref. 9 |
| 172 | 800 | 400 | 375 | 7.797959644 | Late Changhsingian-2 | ref. 9 |
| 173 | 600 | 450 | 425 | 7.778531092 | Late Changhsingian-2 | ref. 9 |
| 174 | 700 | 300 | 275 | 7.480330386 | Late Changhsingian-2 | ref. 9 |
| 175 | 660 | 270 | 255 | 7.376226278 | Late Changhsingian-2 | ref. 9 |
| 176 | 620 | 350 | 325 | 7.567121493 | Late Changhsingian-2 | ref. 9 |
| 177 | 650 | 320 | 300 | 7.513962987 | Late Changhsingian-2 | ref. 9 |
| 178 | 700 | 380 | 365 | 7.705952899 | Late Changhsingian-2 | ref. 9 |
| 179 | 650 | 300 | 280 | 7.45597104 | Late Changhsingian-2 | ref. 9 |
| 180 | 550 | 300 | 275 | 7.375595036 | Late Changhsingian-2 | ref. 9 |
| 181 | 650 | 450 | 425 | 7.813293198 | Late Changhsingian-2 | ref. 9 |
| 182 | 500 | 200 | 185 | 6.985950126 | Late Changhsingian-2 | ref. 9 |
| 183 | 580 | 280 | 265 | 7.352610297 | Late Changhsingian-2 | ref. 9 |
| 184 | 600 | 300 | 275 | 7.413383597 | Late Changhsingian-2 | ref. 9 |
| 185 | 600 | 320 | 305 | 7.486379466 | Late Changhsingian-2 | ref. 9 |
| 186 | 700 | 300 | 275 | 7.480330386 | Late Changhsingian-2 | ref. 9 |
| 187 | 705 | 240 | 210 | 7.269398051 | Late Changhsingian-2 | ref. 9 |
| 188 | 504 | 228 | 192 | 7.06244501 | Late Changhsingian-2 | ref. 9 |
| 189 | 650 | 250 | 225 | 7.281814281 | Late Changhsingian-2 | ref. 9 |
| 190 | 714 | 343 | 314 | 7.604700378 | Late Changhsingian-2 | ref. 9 |
| 191 | 700 | 375 | 337.5 | 7.666181483 | Late Changhsingian-2 | ref. 9 |
| 192 | 660 | 360 | 315 | 7.592935388 | Late Changhsingian-2 | ref. 9 |
| 193 | 690 | 315 | 270 | 7.487301806 | Late Changhsingian-2 | ref. 9 |
| 194 | 505 | 253 | 221 | 7.169582571 | Late Changhsingian-2 | ref. 9 |
| 195 | 650 | 375 | 350 | 7.649791066 | Late Changhsingian-2 | ref. 9 |
| 196 | 675 | 250 | 225 | 7.298204697 | Late Changhsingian-2 | ref. 9 |
| 197 | 675 | 313 | 275 | 6.513636536 | Late Changhsingian-2 | ref. 9 |
| 198 | 625 | 300 | 275 | 7.431112364 | Late Changhsingian-2 | ref. 9 |
| 199 | 700 | 275 | 250 | 7.40114914 | Late Changhsingian-2 | ref. 9 |
| 200 | 690 | 300 | 270 | 7.466112507 | Late Changhsingian-2 | ref. 9 |
| 201 | 600 | 315 | 270 | 7.426603966 | Late Changhsingian-2 | ref. 9 |
| 202 | 540 | 300 | 270 | 7.359657176 | Late Changhsingian-2 | ref. 9 |
| 203 | 645 | 285 | 240 | 7.363394214 | Late Changhsingian-2 | ref. 9 |
| 204 | 570 | 255 | 225 | 6.614215927 | Late Changhsingian-2 | ref. 9 |
| 205 | 660 | 315 | 285 | 7.491477747 | Late Changhsingian-2 | ref. 9 |
| 206 | 630 | 240 | 210 | 7.220549484 | Late Changhsingian-2 | ref. 9 |
| 207 | 615 | 210 | 180 | 7.085145313 | Late Changhsingian-2 | ref. 9 |
| 208 | 660 | 240 | 210 | 7.24075287 | Late Changhsingian-2 | ref. 9 |
| 209 | 347 | 142 | 126 | 6.511766762 | Late Changhsingian-2 | ref. 9 |
| 210 | 411 | 152 | 126 | 6.614834353 | Late Changhsingian-2 | ref. 9 |
| 211 | 720 | 345 | 300 | 7.591051244 | Late Changhsingian-2 | ref. 9 |
| 212 | 615 | 240 | 210 | 7.21008405 | Late Changhsingian-2 | ref. 9 |
| 213 | 450 | 183 | 167 | 6.857158472 | Late Changhsingian-2 | ref. 9 |
| 214 | 450 | 200 | 160 | 6.87714089 | Late Changhsingian-2 | ref. 9 |
| 215 | 650 | 250 | 230 | 7.291359599 | Late Changhsingian-2 | ref. 9 |
| 216 | 300 | 183 | 158 | 6.658713442 | Latest Changhsingian-Earliest Induan | ref. 9 |
| 217 | 300 | 150 | 120 | 6.451172158 | Latest Changhsingian-Earliest Induan | ref. 9 |
| 218 | 555 | 255 | 210 | 7.191830856 | Latest Changhsingian-Earliest Induan | ref. 9 |
| 219 | 480 | 240 | 195 | 7.070265488 | Latest Changhsingian-Earliest Induan | ref. 9 |
| 220 | 400 | 167 | 142 | 6.693954814 | Latest Changhsingian-Earliest Induan | ref. 9 |
| 221 | 433 | 250 | 217 | 7.089332606 | Latest Changhsingian-Earliest Induan | ref. 9 |
| 222 | 690 | 300 | 240 | 7.414959985 | Latest Changhsingian-Earliest Induan | ref. 9 |
| 223 | 400 | 217 | 167 | 6.878479241 | Latest Changhsingian-Earliest Induan | ref. 9 |
| 224 | 545 | 182 | 145 | 6.877901571 | Latest Changhsingian-Earliest Induan | ref. 9 |
| 225 | 460 | 200 | 160 | 6.886686208 | Latest Changhsingian-Earliest Induan | ref. 9 |
| 226 | 420 | 160 | 120 | 6.625328917 | Latest Changhsingian-Earliest Induan | ref. 9 |
| 227 | 541 | 259 | 224 | 7.214455734 | Latest Changhsingian-Earliest Induan | ref. 9 |
| 228 | 460 | 180 | 140 | 6.78293677 | Latest Changhsingian-Earliest Induan | ref. 9 |
| 229 | 480 | 260 | 220 | 7.157415664 | Latest Changhsingian-Earliest Induan | ref. 9 |
| 230 | 480 | 200 | 160 | 6.905169613 | Latest Changhsingian-Earliest Induan | ref. 9 |
| 231 | 500 | 240 | 200 | 7.098989639 | Latest Changhsingian-Earliest Induan | ref. 9 |
| 232 | 360 | 180 | 140 | 6.676481439 | Latest Changhsingian-Earliest Induan | ref. 9 |
| 233 | 300 | 200 | 200 | 6.797959644 | Latest Changhsingian-Earliest Induan | ref. 9 |
| 234 | 300 | 180 | 180 | 6.706444663 | Latest Changhsingian-Earliest Induan | ref. 9 |
| 235 | 250 | 150 | 150 | 6.468900924 | Latest Changhsingian-Earliest Induan | ref. 9 |
| 236 | 500 | 220 | 205 | 7.071924944 | Latest Changhsingian-Earliest Induan | ref. 9 |
| 237 | 420 | 170 | 155 | 6.762808308 | Latest Changhsingian-Earliest Induan | ref. 9 |
| 238 | 500 | 150 | 120 | 6.673020907 | Latest Changhsingian-Earliest Induan | ref. 9 |
| 239 | 300 | 175 | 125 | 6.535847714 | Latest Changhsingian-Earliest Induan | ref. 9 |
| 240 | 520 | 220 | 180 | 7.032476927 | Latest Changhsingian-Earliest Induan | ref. 9 |
| 241 | 467 | 150 | 117 | 6.630823227 | Latest Changhsingian-Earliest Induan | ref. 9 |
| 242 | 600 | 236 | 171 | 7.103398758 | Latest Changhsingian-Earliest Induan | ref. 9 |
| 243 | 557 | 257 | 214 | 7.205912649 | Latest Changhsingian-Earliest Induan | ref. 9 |
| 244 | 483 | 200 | 167 | 6.92590389 | Latest Changhsingian-Earliest Induan | ref. 9 |
| 245 | 600 | 231 | 185 | 7.12637544 | Latest Changhsingian-Earliest Induan | ref. 9 |
| 246 | 643 | 214 | 171 | 7.091969297 | Latest Changhsingian-Earliest Induan | ref. 9 |
| 247 | 415 | 185 | 154 | 6.790583338 | Latest Changhsingian-Earliest Induan | ref. 9 |
| 248 | 669 | 208 | 162 | 7.070050652 | Latest Changhsingian-Earliest Induan | ref. 9 |
| 249 | 429 | 171 | 129 | 6.694029288 | Latest Changhsingian-Earliest Induan | ref. 9 |
| 250 | 357 | 186 | 129 | 6.649610148 | Latest Changhsingian-Earliest Induan | ref. 9 |
| 251 | 627 | 218 | 164 | 7.06893318 | Latest Changhsingian-Earliest Induan | ref. 9 |
| 252 | 398 | 150 | 90 | 6.448995238 | Latest Changhsingian-Earliest Induan | ref. 9 |
| 253 | 596 | 250 | 198 | 7.188629856 | Latest Changhsingian-Earliest Induan | ref. 9 |
| 254 | 520 | 200 | 165 | 6.953295681 | Latest Changhsingian-Earliest Induan | ref. 9 |
| 255 | 600 | 220 | 185 | 7.106524057 | Latest Changhsingian-Earliest Induan | ref. 9 |
| 256 | 350 | 200 | 165 | 6.781360382 | Latest Changhsingian-Earliest Induan | ref. 9 |
| 257 | 400 | 200 | 165 | 6.839352329 | Latest Changhsingian-Earliest Induan | ref. 9 |
| 258 | 500 | 250 | 210 | 7.137907705 | Latest Changhsingian-Earliest Induan | ref. 9 |
| 259 | 650 | 270 | 235 | 7.334123381 | Latest Changhsingian-Earliest Induan | ref. 9 |
| 260 | 450 | 240 | 205 | 7.063956014 | Latest Changhsingian-Earliest Induan | ref. 9 |
| 261 | 600 | 200 | 165 | 7.015443588 | Latest Changhsingian-Earliest Induan | ref. 9 |
| 262 | 700 | 350 | 305 | 7.592244321 | Latest Changhsingian-Earliest Induan | ref. 9 |
| 263 | 500 | 100 | 85 | 6.347167328 | Latest Changhsingian-Earliest Induan | ref. 9 |
| 264 | 350 | 120 | 100 | 6.342027688 | Latest Changhsingian-Earliest Induan | ref. 9 |
| 265 | 400 | 150 | 120 | 6.576110894 | Latest Changhsingian-Earliest Induan | ref. 9 |
| 266 | 400 | 200 | 165 | 6.839352329 | Latest Changhsingian-Earliest Induan | ref. 9 |
| 267 | 420 | 170 | 135 | 6.702810378 | Latest Changhsingian-Earliest Induan | ref. 9 |
| 268 | 600 | 150 | 135 | 6.803354676 | Latest Changhsingian-Earliest Induan | ref. 9 |
| 269 | 300 | 150 | 120 | 6.451172158 | Latest Changhsingian-Earliest Induan | ref. 9 |
| 270 | 550 | 250 | 230 | 7.218808932 | Latest Changhsingian-Earliest Induan | ref. 9 |
| 271 | 600 | 200 | 175 | 7.040997692 | Latest Changhsingian-Earliest Induan | ref. 9 |
| 272 | 420 | 180 | 165 | 6.814784137 | Latest Changhsingian-Earliest Induan | ref. 9 |
| 273 | 500 | 200 | 175 | 6.961816446 | Latest Changhsingian-Earliest Induan | ref. 9 |
| 274 | 600 | 250 | 225 | 7.247052175 | Latest Changhsingian-Earliest Induan | ref. 9 |
| 275 | 603 | 275 | 220 | 7.280851084 | Latest Changhsingian-Earliest Induan | ref. 9 |
| 276 | 498 | 200 | 140 | 6.863165772 | Latest Changhsingian-Earliest Induan | ref. 9 |
| 277 | 605 | 260 | 212 | 7.241842981 | Latest Changhsingian-Earliest Induan | ref. 9 |
| 278 | 740 | 255 | 215 | 7.326988758 | Latest Changhsingian-Earliest Induan | ref. 9 |
| 279 | 738 | 250 | 200 | 7.285804764 | Latest Changhsingian-Earliest Induan | ref. 9 |
| 280 | 600 | 150 | 98 | 6.664246983 | Latest Changhsingian-Earliest Induan | ref. 9 |
| 281 | 255 | 100 | 55 | 5.865681268 | Latest Changhsingian-Earliest Induan | ref. 9 |
| 282 | 500 | 250 | 198 | 7.112353601 | Latest Changhsingian-Earliest Induan | ref. 9 |
| 283 | 550 | 250 | 190 | 7.135834697 | Latest Changhsingian-Earliest Induan | ref. 9 |
| 284 | 500 | 200 | 148 | 6.889040113 | Latest Changhsingian-Earliest Induan | ref. 9 |
| 285 | 600 | 288 | 240 | 7.336533378 | Spathian | ref. 9 |
| 286 | 576 | 288 | 240 | 7.318804611 | Spathian | ref. 9 |
| 287 | 864 | 408 | 336 | 7.792291581 | Spathian | ref. 9 |
| 288 | 912 | 433 | 360 | 7.871563633 | Spathian | ref. 9 |
| 289 | 720 | 360 | 240 | 7.512624637 | Spathian | ref. 9 |
| 290 | 648 | 312 | 192 | 7.307809226 | Spathian | ref. 9 |
| 291 | 528 | 264 | 192 | 7.146317476 | Spathian | ref. 9 |
| 292 | 600 | 240 | 216 | 7.211594641 | Spathian | ref. 9 |
| 293 | 672 | 264 | 264 | 7.389355524 | Spathian | ref. 9 |
| 294 | 672 | 264 | 264 | 7.389355524 | Spathian | ref. 9 |
| 295 | 624 | 288 | 216 | 7.307809226 | Spathian | ref. 9 |
| 296 | 648 | 264 | 216 | 7.286411082 | Spathian | ref. 9 |
| 297 | 312 | 168 | 144 | 6.596604766 | Spathian | ref. 9 |
| 298 | 408 | 120 | 96 | 6.39089104 | Spathian | ref. 9 |
| 299 | 650 | 290 | 240 | 7.374300994 | Spathian | ref. 9 |
| 300 | 620 | 280 | 190 | 7.237081719 | Spathian | ref. 9 |
| 301 | 620 | 260 | 190 | 7.204897036 | Spathian | ref. 9 |
| 302 | 640 | 300 | 240 | 7.382290868 | Spathian | ref. 9 |
| 303 | 790 | 420 | 320 | 7.744804758 | Spathian | ref. 9 |
| 304 | 780 | 380 | 310 | 7.682018291 | Spathian | ref. 9 |
| 305 | 670 | 300 | 240 | 7.402185697 | Spathian | ref. 9 |
| 306 | 540 | 240 | 190 | 7.110137 | Spathian | ref. 9 |
| 307 | 740 | 350 | 370 | 7.700279886 | Spathian | ref. 9 |
| 308 | 750 | 380 | 310 | 7.664984952 | Spathian | ref. 9 |
| 309 | 770 | 400 | 420 | 7.830578405 | Spathian | ref. 9 |
| 310 | 790 | 410 | 420 | 7.852438636 | Spathian | ref. 9 |
| 311 | 780 | 350 | 370 | 7.723142769 | Spathian | ref. 9 |
| 312 | 850 | 460 | 460 | 7.973712987 | Spathian | ref. 9 |
| 313 | 650 | 380 | 250 | 7.50941536 | Spathian | ref. 9 |
| 314 | 500 | 350 | 240 | 7.342027688 | Spathian | ref. 9 |
| 315 | 500 | 260 | 250 | 7.230661759 | Spathian | ref. 9 |
| 316 | 420 | 220 | 180 | 6.939722874 | Spathian | ref. 9 |
| 317 | 860 | 430 | 250 | 7.684685313 | Spathian | ref. 9 |

Table S4. **Stable isotopic compositions of soil carbonates from northern Shanxi, North China.**

| Samples | ^13^C | ^18^O |
| --- | --- | --- |
| BD60-1 | -3.1 | -10.1 |
| BD59-1 | -3.3 | -8.4 |
| BD57-3 | -3.2 | -7.1 |
| BD57-2 | -3.0 | -8.4 |
| BD57-1 | -2.5 | -8.0 |
| BD56-3 | -2.9 | -8.3 |
| BD56-2 | -2.9 | -4.2 |
| BD56-1 | -2.8 | -10.0 |
| BD55-2 | -2.5 | -10.6 |
| BD55-1 | -2.6 | -12.2 |
| BD54-6 | -2.6 | -9.2 |
| BD54-5 | -3.0 | -8.8 |
| BD54-4 | -2.9 | -8.9 |
| BD54-3 | -3.1 | -11.9 |
| BD54-2 | -3.2 | -10.7 |
| BD54-1 | -3.0 | -12.4 |
| BD53-1 | -3.3 | -11.2 |
| BD52-4 | -3.7 | -7.7 |
| BD52-3 | -5.3 | -8.2 |
| BD51-4 | -6.0 | -10.7 |
| BD51-3 | -4.9 | -10.6 |
| BD51-2 | -3.7 | -12.3 |
| BD51-1 | -4.8 | -11.5 |
| BD49-1 | -4.1 | -12.4 |
| BD48-1 | -4.5 | -11.4 |
| BD45-2 | -4.3 | -9.9 |
| BD45-1 | -5.1 | -8.9 |
| BD40-2 | -4.0 | -8.1 |
| BD40-1 | -3.1 | -10.9 |
| BD39-2 | -2.6 | -10.3 |
| BD39-1 | -1.7 | -10.2 |
| BD33-3 | -5.9 | -8.8 |
| BD29-3 | -3.2 | -9.9 |
| BD29-2 | -5.8 | -10.0 |
| BD29-1 | -3.4 | -8.6 |
| BD28-1 | -4.8 | -9.0 |
| BD11-1 | -6.9 | -9.9 |
| BD10-1 | -3.3 | -13.2 |
| BD08-1 | -7.5 | -9.9 |
| BD06-1 | -7.8 | -9.6 |
| BD05-1 | -3.1 | -13.3 |
| DZ34-1 | -1.5 | -9.5 |
| DZ27-1 | -1.7 | -9.5 |
| DZ13-1 | -2.9 | -10.8 |
| DZ09-1 | -3.2 | -13.7 |
| DZ08-1 | -2.4 | -13.9 |
| DZ07-1 | -3.8 | -12.8 |
| DZ05-1 | -3.2 | -7.8 |

Table S5. **Geochemical proxy profiles of mudstones from northern Shanxi, North China.**

| Samples | CO_2_(%) | CIA | *W | Ba/Sr | （K+Na)/Al | Rb/K(10^-3^) | V/Cr | Sr/Ca | Mg/Ca | Ba/Ca | Al/Si | Sc/Zr | Lu/Hf | Mn/Sr |
| --- | --- | --- | --- | --- | --- | --- | --- | --- | --- | --- | --- | --- | --- | --- |
| BD60-2 | 0.26 | 66.06 | 9.14 | 2.33 | 0.45 | 3.39 | 0.79 | 0.03 | 4.67 | 0.08 | 0.29 | 0.05 | 0.07 | 1.25 |
| BD60-1 | 2.58 | 53.47 | -3.45 | 1.83 | 0.57 | 3.25 | 0.69 | 0.01 | 0.75 | 0.03 | 0.26 | 0.05 | 0.06 | 0.99 |
| BD59-2 | 0.34 | 63.16 | 6.24 | 1.74 | 0.51 | 3.22 | 1.15 | 0.04 | 4.53 | 0.07 | 0.28 | 0.05 | 0.06 | 0.95 |
| BD59-1 | 0.34 | 65.51 | 8.59 | 1.92 | 0.44 | 4.7 | 1.12 | 0.03 | 4.55 | 0.06 | 0.3 | 0.08 | 0.07 | 1.31 |
| BD57-3 | 0.77 | 65.44 | 8.52 | 1.48 | 0.4 | 3.84 | 2.36 | 0.02 | 2.82 | 0.02 | 0.34 | 0.1 | 0.12 | 3.31 |
| BD57-2 | 4.03 | 52.32 | -4.6 | 1.5 | 0.49 | 4.29 | 1.22 | 0.01 | 0.66 | 0.01 | 0.29 | 0.05 | 0.05 | 1.68 |
| BD57-1 | 7.97 | 45.15 | -11.77 | 1.18 | 0.44 | 4.71 | 1.15 | 0 | 0.39 | 0.01 | 0.3 | 0.06 | 0.07 | 3.05 |
| BD56-3 | 9.35 | 44.88 | -12.04 | 1.21 | 0.35 | 5.35 | 1.69 | 0 | 0.37 | 0 | 0.34 | 0.13 | 0.11 | 3.9 |
| BD56-2 | 0.94 | 68.55 | 11.63 | 1.43 | 0.33 | 4.35 | 1.66 | 0.01 | 2.65 | 0.02 | 0.36 | 0.12 | 0.09 | 2.49 |
| BD56-1 | 7.28 | 46.72 | -10.2 | 1.37 | 0.45 | 4.39 | 1.19 | 0.01 | 0.44 | 0.01 | 0.31 | 0.13 | 0.09 | 2.68 |
| BD55-2 | 3.25 | 57.22 | 0.3 | 1.53 | 0.42 | 4.58 | 2.3 | 0.01 | 0.96 | 0.01 | 0.32 | 0.1 | 0.09 | 2.45 |
| BD55-1 | 1.46 | 63.11 | 6.19 | 1.32 | 0.41 | 3.83 | 2.07 | 0.01 | 1.8 | 0.02 | 0.34 | 0.12 | 0.09 | 2.37 |
| BD54-6 | 7.71 | 46.11 | -10.81 | 1.18 | 0.44 | 4.75 | 1.64 | 0.01 | 0.43 | 0.01 | 0.32 | 0.1 | 0.09 | 2.94 |
| BD54-5 | 7.03 | 48.2 | -8.72 | 1.16 | 0.41 | 4.96 | 1.65 | 0.01 | 0.52 | 0.01 | 0.33 | 0.1 | 0.09 | 2.75 |
| BD54-4 | 1.88 | 60.87 | 3.95 | 1.2 | 0.42 | 3.26 | 1.47 | 0.01 | 1.45 | 0.01 | 0.3 | 0.07 | 0.08 | 2 |
| BD54-3 | 1.46 | 59.6 | 2.68 | 1.4 | 0.47 | 3.66 | 1.08 | 0.02 | 1.22 | 0.02 | 0.26 | 0.05 | 0.06 | 1.27 |
| BD54-2 | 4.45 | 49.98 | -6.94 | 1.33 | 0.46 | 4.22 | 1.28 | 0.01 | 0.49 | 0.01 | 0.26 | 0.05 | 0.07 | 2.06 |
| BD54-1 | 2.58 | 54.02 | -2.9 | 1.47 | 0.53 | 3.85 | 1.11 | 0.01 | 0.66 | 0.02 | 0.23 | 0.04 | 0.05 | 1.12 |
| BD53-1 | 9.35 | 38.73 | -18.19 | 1.17 | 0.47 | 4.42 | 1.09 | 0 | 0.33 | 0.01 | 0.28 | 0.05 | 0.07 | 2.9 |
| BD52-4 | 5.4 | 49.25 | -7.67 | 1.05 | 0.48 | 3.93 | 0.98 | 0.01 | 0.55 | 0.01 | 0.28 | 0.04 | 0.07 | 1.19 |
| BD52-3 | 1.46 | 60.33 | 3.41 | 1.2 | 0.45 | 4.44 | 1.06 | 0.02 | 1.66 | 0.02 | 0.31 | 0.07 | 0.07 | 1.1 |
| BD52-2 | 0.09 | 69.49 | 12.57 | 0.98 | 0.36 | 4 | 1.09 | 0.05 | 4.47 | 0.05 | 0.35 | 0.04 | 0.06 | 0.72 |
| BD52-1 | 0.17 | 62.51 | 5.59 | 1.28 | 0.51 | 3.69 | 1.24 | 0.05 | 3.22 | 0.06 | 0.29 | 0.06 | 0.06 | 0.93 |
| BD51-4 | 0.26 | 60.06 | 3.14 | 1.45 | 0.55 | 2.51 | 1.1 | 0.04 | 2.21 | 0.06 | 0.27 | 0.03 | 0.03 | 0.73 |
| BD51-3 | 0.17 | 60.77 | 3.85 | 1.49 | 0.55 | 3.05 | 1.02 | 0.05 | 2.73 | 0.07 | 0.28 | 0.05 | 0.06 | 0.65 |
| BD51-2 | 2.74 | 52 | -4.92 | 1.3 | 0.59 | 3.03 | 1.36 | 0.02 | 0.65 | 0.02 | 0.27 | 0.03 | 0.04 | 0.95 |
| BD51-1 | 0.09 | 61.12 | 4.2 | 1.4 | 0.54 | 3.45 | 1.28 | 0.05 | 2.84 | 0.07 | 0.29 | 0.04 | 0.03 | 0.61 |
| BD49-1 | 3.35 | 51.8 | -5.12 | 2.34 | 0.56 | 3.34 | 0.99 | 0.01 | 0.59 | 0.02 | 0.26 | 0.02 | 0.03 | 1.58 |
| BD45-2 | 8.23 | 45.77 | -11.15 | 1.46 | 0.42 | 4.59 | 1.16 | 0 | 0.45 | 0.01 | 0.33 | 0.1 | 0.08 | 3.23 |
| BD45-1 | 0.86 | 60.92 | 4 | 1.31 | 0.49 | 2.47 | 0.81 | 0.02 | 1.79 | 0.03 | 0.3 | 0.06 | 0.06 | 1.08 |
| BD40-2 | 6.17 | 49.81 | -7.11 | 1.35 | 0.44 | 4.18 | 1.01 | 0.01 | 0.56 | 0.01 | 0.33 | 0.07 | 0.06 | 2.3 |
| BD40-1 | 0.78 | 64.05 | 7.13 | 1.01 | 0.44 | 3.24 | 1.02 | 0.03 | 2.57 | 0.03 | 0.31 | 0.07 | 0.07 | 1 |
| BD39-2 | 3.43 | 58.86 | 1.94 | 1.05 | 0.38 | 4.09 | 1.6 | 0.01 | 1.01 | 0.01 | 0.34 | 0.09 | 0.09 | 2.25 |
| BD39-1 | 1.54 | 62.81 | 5.89 | 1.2 | 0.4 | 4.72 | 1.07 | 0.02 | 1.82 | 0.02 | 0.32 | 0.07 | 0.08 | 1.26 |
| BD35-1 | 0.26 | 68.06 | 11.14 | 1.78 | 0.4 | 3.4 | 1.14 | 0.04 | 5.35 | 0.07 | 0.36 | 0.07 | 0.09 | 1.92 |
| BD33-3 | 0.17 | 64.84 | 7.92 | 1.76 | 0.47 | 3.69 | 0.69 | 0.04 | 3.6 | 0.07 | 0.3 | 0.05 | 0.05 | 0.78 |
| BD33-2 | 0.26 | 68.36 | 11.44 | 2.13 | 0.39 | 4.02 | 0.81 | 0.03 | 3.99 | 0.06 | 0.32 | 0.07 | 0.11 | 1.8 |
| BD33-1 | 0.26 | 62.96 | 6.04 | 1.46 | 0.51 | 3.58 | 0.57 | 0.06 | 2.91 | 0.08 | 0.27 | 0.06 | 0.06 | 0.66 |
| BD29-3 | 1.98 | 58.3 | 1.38 | 2.38 | 0.35 | 2.96 | 1.08 | 0.01 | 2.33 | 0.03 | 0.37 | 0.09 | 0.22 | 2.99 |
| BD29-2 | 22.8 | 13.11 | -43.81 | 1.83 | 0.25 | 3.7 | 1.34 | 0 | 0.06 | 0 | 0.27 | 0.1 | 0.09 | 1.68 |
| BD29-1 | 2.4 | 52.69 | -4.23 | 0.97 | 0.26 | 4.05 | 0.96 | 0.01 | 1.41 | 0.01 | 0.28 | 0.07 | 0.07 | 6.48 |
| BD22-1 | 0.09 | 63.42 | 6.5 | 1.39 | 0.49 | 3.31 | 0.91 | 0.05 | 2.85 | 0.07 | 0.3 | 0.03 | 0.04 | 0.94 |
| BD10-1 | 0.17 | 69.02 | 12.1 | 1.42 | 0.38 | 3.63 | 1.4 | 0.05 | 3.29 | 0.07 | 0.32 | 0.06 | 0.08 | 1.85 |
| BD05-1 | 0.17 | 65.05 | 8.13 | 1.66 | 0.47 | 4.01 | 1.04 | 0.06 | 2.24 | 0.1 | 0.28 | 0.05 | 0.05 | 1.24 |
| DZ45-1 | 0.09 | 66.08 | 5.93 | 4.66 | 0.44 | 1.88 | 0.83 | 1.95 | 1.89 | 0.09 | 0.29 | 0.05 | 0.07 | 2.43 |
| DZ38-1 | 0.09 | 60.23 | 0.08 | 2.46 | 0.57 | 1.49 | 0.56 | 3.65 | 0.98 | 0.09 | 0.25 | 0.03 | 0.04 | 0.71 |
| DZ34-1 | 0.26 | 62.86 | 2.71 | 2.85 | 0.47 | 1.61 | 0.85 | 2.13 | 1.08 | 0.06 | 0.28 | 0.02 | 0.03 | 1.58 |
| DZ27-1 | 0.09 | 61.71 | 1.56 | 2.78 | 0.54 | 1.41 | 0.79 | 3.67 | 1.29 | 0.1 | 0.28 | 0.01 | 0.03 | 0.93 |
| DZ26-1 | 0.09 | 65.14 | 4.99 | 3.74 | 0.48 | 1.54 | 0.77 | 3.06 | 2.09 | 0.11 | 0.39 | 0.04 | 0.06 | 1.35 |
| DZ14-1 | 0.09 | 62.62 | 2.47 | 2.05 | 0.51 | 1.11 | 0.96 | 2.87 | 1.61 | 0.06 | 0.26 | 0.01 | 0.03 | 1.22 |
| DZ13-1 | 1.97 | 55.08 | -5.07 | 1.63 | 0.49 | 1.58 | 0.96 | 1.41 | 0.35 | 0.02 | 0.24 | 0.02 | 0.04 | 1.34 |
| DZ10-1 | 0.17 | 64.2 | 4.05 | 3.02 | 0.49 | 1.75 | 0.64 | 3.11 | 2.19 | 0.09 | 0.26 | 0.05 | 0.06 | 1.21 |
| DZ09-1 | 1.11 | 55.48 | -4.67 | 2.32 | 0.63 | 1.43 | 0.8 | 2.05 | 0.63 | 0.05 | 0.2 | 0.05 | 0.07 | 0.69 |
| DZ08-1 | 0.78 | 57.16 | -2.99 | 2.42 | 0.59 | 1.47 | 0.83 | 2.31 | 0.7 | 0.06 | 0.21 | 0.03 | 0.04 | 0.8 |
| DZ07-1 | 4.63 | 45.84 | -14.31 | 1.89 | 0.66 | 1.35 | 1.11 | 0.99 | 0.16 | 0.02 | 0.22 | 0.03 | 0.04 | 0.91 |
| DZ05-1 | 1.37 | 65.44 | 5.29 | 3.53 | 0.38 | 1.44 | 1.19 | 0.66 | 1.71 | 0.02 | 0.37 | 0.1 | 0.14 | 4.46 |

Table 6. **U–Pb isotopic analyses of detrital zircons in the Sunjiagou and Liujiagou Foramations from northern Shanxi, North China.**

| **spot** | **No.** | **Measured isotopic ratios** | | | | | | | **Corrected isotopic ages** | | | | | | **Used age** | | **Concordance** |
| --- | --- | --- | --- | --- | --- | --- | --- | --- | --- | --- | --- | --- | --- | --- | --- | --- | --- |
|  | **sample** | **^207^Pb/^206^Pb** | **1σ** | **^207^Pb/^235^U** | **1σ** | **^206^Pb/^238^U** | **1σ** | **^232^Th/^238^U** | **^207^Pb/^206^Pb** | **1σ** | **^207^Pb/^235^U** | **1σ** | **^206^Pb/^238^U** | **1σ** |  |  |  |
| **Liujiagou Formation, sample 2015BDLJG-1, GPS: 111° 00′ 51.87″ E, 39° 00′ 07.75″ N** | | | | | | | | | | | | | | | | | |
| 1 | 46 | 0.0550 | ~~0.0079~~ | ~~0.2783~~ | ~~0.0403~~ | ~~0.0366~~ | ~~0.0019~~ | ~~1.1819~~ | ~~409~~ | ~~325~~ | ~~249~~ | ~~32~~ | ~~231~~ | ~~12~~ | **~~231~~** | **~~12~~** | ~~92%~~ |
| 2 | 58 | 0.0508 | 0.0032 | 0.2783 | 0.0185 | 0.0397 | 0.0007 | 0.8988 | 235 | 146 | 249 | 15 | 251 | 4 | **251** | **4** | 99% |
| 3 | 64 | 0.0518 | 0.0021 | 0.3136 | 0.0134 | 0.0440 | 0.0006 | 1.3750 | 276 | 90 | 277 | 10 | 278 | 4 | **278** | **4** | 99% |
| 4 | 75 | 0.0558 | 0.0054 | 0.3465 | 0.0289 | 0.0481 | 0.0015 | 0.7142 | 456 | 219 | 302 | 22 | 303 | 9 | **303** | **9** | 99% |
| 5 | 77 | 0.0567 | 0.0067 | 0.3671 | 0.0396 | 0.0483 | 0.0012 | 1.3801 | 480 | 261 | 317 | 29 | 304 | 8 | **304** | **8** | 95% |
| 6 | 76 | 0.0556 | 0.0022 | 0.3667 | 0.0133 | 0.0484 | 0.0006 | 0.7035 | 439 | 89 | 317 | 10 | 305 | 4 | **305** | **4** | 95% |
| 7 | 40 | 0.0513 | 0.0018 | 0.3415 | 0.0117 | 0.0485 | 0.0005 | 0.7279 | 254 | 81 | 298 | 9 | 305 | 3 | **305** | **3** | 97% |
| 8 | 84 | 0.0538 | 0.0029 | 0.3582 | 0.0193 | 0.0485 | 0.0007 | 1.0424 | 361 | 122 | 311 | 14 | 305 | 4 | **305** | **4** | 98% |
| 9 | 78 | 0.0497 | 0.0026 | 0.3279 | 0.0168 | 0.0486 | 0.0008 | 1.4170 | 189 | 124 | 288 | 13 | 306 | 5 | **306** | **5** | 93% |
| 10 | 34 | 0.0557 | 0.0014 | 0.3743 | 0.0090 | 0.0487 | 0.0005 | 1.0314 | 443 | 54 | 323 | 7 | 307 | 3 | **307** | **3** | 94% |
| 11 | 18 | 0.0579 | 0.0030 | 0.3859 | 0.0194 | 0.0489 | 0.0011 | 0.5756 | 528 | 115 | 331 | 14 | 308 | 7 | **308** | **7** | 92% |
| ~~12~~ | ~~24~~ | ~~0.0664~~ | ~~0.0042~~ | ~~0.4373~~ | ~~0.0254~~ | ~~0.0489~~ | ~~0.0010~~ | ~~0.9056~~ | ~~820~~ | ~~133~~ | ~~368~~ | ~~18~~ | ~~308~~ | ~~6~~ | **~~308~~** | **~~6~~** | ~~82%~~ |
| 13 | 62 | 0.0544 | 0.0048 | 0.3453 | 0.0250 | 0.0489 | 0.0022 | 1.2365 | 391 | 200 | 301 | 19 | 308 | 13 | **308** | **13** | 97% |
| 14 | 12 | 0.0589 | 0.0035 | 0.3955 | 0.0239 | 0.0491 | 0.0010 | 0.8123 | 565 | 128 | 338 | 17 | 309 | 6 | **309** | **6** | 90% |
| 15 | 61 | 0.0548 | 0.0033 | 0.3696 | 0.0204 | 0.0492 | 0.0008 | 0.5477 | 406 | 169 | 319 | 15 | 310 | 5 | **310** | **5** | 96% |
| 16 | 27 | 0.0513 | 0.0016 | 0.3478 | 0.0108 | 0.0492 | 0.0006 | 0.8369 | 254 | 68 | 303 | 8 | 310 | 3 | **310** | **3** | 97% |
| 17 | 19 | 0.0569 | 0.0025 | 0.3837 | 0.0170 | 0.0493 | 0.0006 | 0.5885 | 487 | 98 | 330 | 12 | 310 | 4 | **310** | **4** | 93% |
| 18 | 22 | 0.0533 | 0.0025 | 0.3581 | 0.0167 | 0.0493 | 0.0006 | 0.5173 | 343 | 112 | 311 | 12 | 310 | 4 | **310** | **4** | 99% |
| 19 | 44 | 0.0590 | 0.0030 | 0.3974 | 0.0208 | 0.0495 | 0.0007 | 0.6973 | 565 | 113 | 340 | 15 | 312 | 4 | **312** | **4** | 91% |
| 20 | 48 | 0.0600 | 0.0040 | 0.3916 | 0.0210 | 0.0495 | 0.0009 | 0.4435 | 611 | 144 | 336 | 15 | 312 | 5 | **312** | **5** | 92% |
| 21 | 30 | 0.0599 | 0.0029 | 0.4026 | 0.0193 | 0.0503 | 0.0016 | 1.7998 | 598 | 101 | 344 | 14 | 316 | 10 | **316** | **10** | 91% |
| 22 | 59 | 0.0492 | 0.0024 | 0.3932 | 0.0189 | 0.0581 | 0.0007 | 0.9641 | 167 | 149 | 337 | 14 | 364 | 4 | **364** | **4** | 92% |
| 23 | 13 | 0.0546 | 0.0015 | 0.4515 | 0.0115 | 0.0602 | 0.0005 | 0.6506 | 398 | 56 | 378 | 8 | 377 | 3 | **377** | **3** | 99% |
| 24 | 79 | 0.0552 | 0.0031 | 0.4827 | 0.0280 | 0.0638 | 0.0010 | 0.5149 | 420 | 119 | 400 | 19 | 399 | 6 | **399** | **6** | 99% |
| 25 | 25 | 0.0564 | 0.0031 | 0.4858 | 0.0244 | 0.0639 | 0.0011 | 0.6620 | 478 | 122 | 402 | 17 | 399 | 7 | **399** | **7** | 99% |
| 26 | 85 | 0.0518 | 0.0030 | 0.4570 | 0.0256 | 0.0644 | 0.0009 | 0.5900 | 276 | 103 | 382 | 18 | 402 | 5 | **402** | **5** | 94% |
| 27 | 10 | 0.0601 | 0.0025 | 0.5679 | 0.0226 | 0.0690 | 0.0008 | 0.5822 | 607 | 86 | 457 | 15 | 430 | 5 | **430** | **5** | 94% |
| 28 | 49 | 0.0586 | 0.0026 | 0.5682 | 0.0225 | 0.0725 | 0.0013 | 0.5519 | 550 | 103 | 457 | 15 | 451 | 8 | **451** | **8** | 98% |
| ~~29~~ | ~~56~~ | ~~0.0821~~ | ~~0.0081~~ | ~~0.5887~~ | ~~0.0518~~ | ~~0.0537~~ | ~~0.0013~~ | ~~1.1552~~ | ~~1250~~ | ~~194~~ | ~~470~~ | ~~33~~ | ~~337~~ | ~~8~~ | **~~1250~~** | **~~194~~** | ~~67%~~ |
| ~~30~~ | ~~65~~ | ~~0.0839~~ | ~~0.0066~~ | ~~3.2546~~ | ~~0.2474~~ | ~~0.2839~~ | ~~0.0076~~ | ~~0.5635~~ | ~~1300~~ | ~~154~~ | ~~1470~~ | ~~59~~ | ~~1611~~ | ~~38~~ | **~~1300~~** | **~~154~~** | ~~90%~~ |
| 31 | 66 | 0.0912 | 0.0027 | 4.3854 | 0.1281 | 0.3515 | 0.0055 | 0.6942 | 1452 | 56 | 1710 | 24 | 1942 | 26 | **1452** | **56** | 87% |
| 32 | 31 | 0.0968 | 0.0016 | 3.7525 | 0.0649 | 0.2805 | 0.0022 | 0.6142 | 1563 | 31 | 1583 | 14 | 1594 | 11 | **1563** | **31** | 99% |
| 33 | 28 | 0.0976 | 0.0050 | 3.5434 | 0.1847 | 0.2650 | 0.0055 | 0.6515 | 1589 | 96 | 1537 | 41 | 1515 | 28 | **1589** | **96** | 98% |
| 34 | 83 | 0.0981 | 0.0023 | 3.8433 | 0.0965 | 0.2861 | 0.0047 | 0.5347 | 1591 | 43 | 1602 | 20 | 1622 | 24 | **1591** | **43** | 98% |
| 35 | 7 | 0.1001 | 0.0018 | 3.8353 | 0.0695 | 0.2782 | 0.0027 | 0.4905 | 1626 | 33 | 1600 | 15 | 1582 | 14 | **1626** | **33** | 98% |
| 36 | 42 | 0.1013 | 0.0037 | 3.9082 | 0.1545 | 0.2784 | 0.0044 | 0.5706 | 1648 | 63 | 1615 | 32 | 1583 | 22 | **1648** | **63** | 98% |
| 37 | 29 | 0.1015 | 0.0033 | 3.9704 | 0.1207 | 0.2849 | 0.0042 | 1.1512 | 1654 | 66 | 1628 | 25 | 1616 | 21 | **1654** | **66** | 99% |
| 38 | 68 | 0.1019 | 0.0053 | 3.9162 | 0.2033 | 0.2854 | 0.0084 | 0.6055 | 1658 | 95 | 1617 | 42 | 1619 | 42 | **1658** | **95** | 99% |
| 39 | 36 | 0.1021 | 0.0022 | 3.9925 | 0.0923 | 0.2831 | 0.0037 | 0.5708 | 1662 | 40 | 1633 | 19 | 1607 | 19 | **1662** | **40** | 98% |
| 40 | 39 | 0.1023 | 0.0028 | 3.9501 | 0.1159 | 0.2791 | 0.0032 | 0.5583 | 1666 | 51 | 1624 | 24 | 1587 | 16 | **1666** | **51** | 97% |
| 41 | 8 | 0.1023 | 0.0032 | 3.8424 | 0.1083 | 0.2765 | 0.0047 | 0.4510 | 1666 | 59 | 1602 | 23 | 1574 | 24 | **1666** | **59** | 98% |
| 42 | 41 | 0.1029 | 0.0029 | 3.9242 | 0.1157 | 0.2770 | 0.0045 | 0.9626 | 1677 | 52 | 1619 | 24 | 1576 | 23 | **1677** | **52** | 97% |
| 43 | 72 | 0.1037 | 0.0020 | 4.7209 | 0.0932 | 0.3303 | 0.0032 | 0.6379 | 1692 | 36 | 1771 | 17 | 1840 | 16 | **1692** | **36** | 96% |
| 44 | 80 | 0.1057 | 0.0036 | 4.8414 | 0.1708 | 0.3325 | 0.0049 | 0.7311 | 1728 | 63 | 1792 | 30 | 1850 | 24 | **1728** | **63** | 96% |
| 45 | 82 | 0.1060 | 0.0032 | 4.7705 | 0.1741 | 0.3264 | 0.0059 | 0.9510 | 1731 | 54 | 1780 | 31 | 1821 | 29 | **1731** | **54** | 97% |
| 46 | 16 | 0.1061 | 0.0035 | 4.6810 | 0.1589 | 0.3198 | 0.0043 | 0.7614 | 1733 | 61 | 1764 | 28 | 1789 | 21 | **1733** | **61** | 98% |
| 47 | 54 | 0.1068 | 0.0046 | 4.9166 | 0.2081 | 0.3355 | 0.0055 | 1.0121 | 1746 | 84 | 1805 | 36 | 1865 | 27 | **1746** | **84** | 96% |
| 48 | 37 | 0.1092 | 0.0024 | 4.8275 | 0.1098 | 0.3202 | 0.0041 | 0.6113 | 1787 | 39 | 1790 | 19 | 1791 | 20 | **1787** | **39** | 99% |
| 49 | 14 | 0.1116 | 0.0034 | 5.3835 | 0.1529 | 0.3524 | 0.0048 | 0.6357 | 1825 | 55 | 1882 | 24 | 1946 | 23 | **1825** | **55** | 96% |
| 50 | 52 | 0.1117 | 0.0021 | 5.4240 | 0.1102 | 0.3527 | 0.0040 | 0.8176 | 1828 | 33 | 1889 | 17 | 1948 | 19 | **1828** | **33** | 96% |
| 51 | 1 | 0.1118 | 0.0019 | 5.1491 | 0.0877 | 0.3340 | 0.0030 | 0.3985 | 1829 | 30 | 1844 | 14 | 1858 | 15 | **1829** | **30** | 99% |
| 52 | 70 | 0.1124 | 0.0028 | 5.4672 | 0.1439 | 0.3526 | 0.0069 | 0.4575 | 1839 | 45 | 1895 | 23 | 1947 | 33 | **1839** | **45** | 97% |
| 53 | 38 | 0.1173 | 0.0033 | 5.5782 | 0.1505 | 0.3457 | 0.0046 | 0.9581 | 1917 | 50 | 1913 | 23 | 1914 | 22 | **1917** | **50** | 99% |
| 54 | 6 | 0.1183 | 0.0013 | 5.6244 | 0.0790 | 0.3447 | 0.0040 | 0.3368 | 1931 | 14 | 1920 | 12 | 1909 | 19 | **1931** | **14** | 99% |
| 55 | 55 | 0.1189 | 0.0042 | 5.6829 | 0.1966 | 0.3519 | 0.0071 | 0.6676 | 1940 | 96 | 1929 | 30 | 1944 | 34 | **1940** | **96** | 99% |
| 56 | 4 | 0.1219 | 0.0031 | 5.6453 | 0.1352 | 0.3389 | 0.0047 | 1.2331 | 1984 | 45 | 1923 | 21 | 1881 | 23 | **1984** | **45** | 97% |
| 57 | 45 | 0.1233 | 0.0023 | 6.2023 | 0.1092 | 0.3665 | 0.0041 | 0.7659 | 2006 | 32 | 2005 | 15 | 2013 | 19 | **2006** | **32** | 99% |
| 58 | 33 | 0.1244 | 0.0034 | 6.3019 | 0.1589 | 0.3678 | 0.0047 | 1.3205 | 2020 | 53 | 2019 | 22 | 2019 | 22 | **2020** | **53** | 99% |
| 59 | 11 | 0.1346 | 0.0013 | 7.4861 | 0.0707 | 0.4027 | 0.0022 | 0.3465 | 2158 | 17 | 2171 | 8 | 2182 | 10 | **2158** | **17** | 99% |
| 60 | 69 | 0.1371 | 0.0017 | 8.1180 | 0.1215 | 0.4286 | 0.0037 | 0.9711 | 2190 | 22 | 2244 | 14 | 2299 | 17 | **2190** | **22** | 97% |
| 61 | 57 | 0.1374 | 0.0016 | 8.0923 | 0.1120 | 0.4259 | 0.0037 | 0.5186 | 2195 | 21 | 2241 | 13 | 2287 | 17 | **2195** | **21** | 97% |
| 62 | 73 | 0.1401 | 0.0012 | 8.4716 | 0.0848 | 0.4381 | 0.0033 | 0.6972 | 2228 | 15 | 2283 | 9 | 2342 | 15 | **2228** | **15** | 97% |
| 63 | 67 | 0.1434 | 0.0032 | 8.5723 | 0.1870 | 0.4351 | 0.0048 | 1.7071 | 2269 | 39 | 2294 | 20 | 2329 | 22 | **2269** | **39** | 98% |
| 64 | 71 | 0.1436 | 0.0032 | 8.6121 | 0.1814 | 0.4371 | 0.0057 | 0.6871 | 2272 | 39 | 2298 | 19 | 2337 | 25 | **2272** | **39** | 98% |
| 65 | 63 | 0.1454 | 0.0044 | 8.5790 | 0.2347 | 0.4310 | 0.0091 | 0.6712 | 2292 | 52 | 2294 | 25 | 2310 | 41 | **2292** | **52** | 99% |
| 66 | 32 | 0.1458 | 0.0022 | 8.6779 | 0.1573 | 0.4299 | 0.0045 | 0.5847 | 2298 | 26 | 2305 | 17 | 2305 | 20 | **2298** | **26** | 99% |
| 67 | 53 | 0.1496 | 0.0022 | 9.3095 | 0.1381 | 0.4518 | 0.0037 | 0.6782 | 2343 | 25 | 2369 | 14 | 2403 | 17 | **2343** | **25** | 98% |
| 68 | 2 | 0.1507 | 0.0021 | 9.1155 | 0.1224 | 0.4386 | 0.0031 | 1.7197 | 2354 | 23 | 2350 | 12 | 2344 | 14 | **2354** | **23** | 99% |
| 69 | 20 | 0.1515 | 0.0041 | 9.4096 | 0.2214 | 0.4519 | 0.0055 | 0.7721 | 2363 | 46 | 2379 | 22 | 2404 | 24 | **2363** | **46** | 98% |
| 70 | 17 | 0.1532 | 0.0021 | 9.3981 | 0.1340 | 0.4445 | 0.0032 | 0.9853 | 2381 | 24 | 2378 | 13 | 2371 | 14 | **2381** | **24** | 99% |
| 71 | 74 | 0.1533 | 0.0053 | 9.5501 | 0.3689 | 0.4504 | 0.0078 | 0.6910 | 2384 | 59 | 2392 | 36 | 2397 | 35 | **2384** | **59** | 99% |
| 72 | 50 | 0.1536 | 0.0025 | 9.1821 | 0.1792 | 0.4337 | 0.0052 | 1.0937 | 2387 | 27 | 2356 | 18 | 2322 | 24 | **2387** | **27** | 98% |
| 73 | 51 | 0.1553 | 0.0026 | 10.0579 | 0.1656 | 0.4702 | 0.0043 | 0.3455 | 2405 | 29 | 2440 | 15 | 2484 | 19 | **2405** | **29** | 98% |
| 74 | 60 | 0.1565 | 0.0030 | 10.0798 | 0.2120 | 0.4675 | 0.0091 | 0.4513 | 2418 | 32 | 2442 | 19 | 2473 | 40 | **2418** | **32** | 98% |
| 75 | 81 | 0.1568 | 0.0035 | 10.1167 | 0.2366 | 0.4721 | 0.0094 | 1.0429 | 2421 | 38 | 2445 | 22 | 2493 | 41 | **2421** | **38** | 98% |
| 76 | 3 | 0.1598 | 0.0015 | 10.1974 | 0.1157 | 0.4630 | 0.0043 | 0.6821 | 2453 | 15 | 2453 | 10 | 2453 | 19 | **2453** | **15** | 99% |
| 77 | 88 | 0.1596 | 0.0046 | 10.2451 | 0.3427 | 0.4651 | 0.0089 | 0.7363 | 2454 | 49 | 2457 | 31 | 2462 | 39 | **2454** | **49** | 99% |
| 78 | 21 | 0.1603 | 0.0040 | 10.3265 | 0.2652 | 0.4700 | 0.0081 | 0.7337 | 2459 | 42 | 2464 | 24 | 2484 | 35 | **2459** | **42** | 99% |
| 79 | 47 | 0.1604 | ~~0.0019~~ | ~~10.2546~~ | ~~0.1522~~ | ~~0.4629~~ | ~~0.0043~~ | ~~0.5446~~ | ~~2460~~ | ~~20~~ | ~~2458~~ | ~~14~~ | ~~2452~~ | ~~19~~ | **2460** | **20** | ~~99%~~ |
| 80 | 43 | 0.1608 | 0.0020 | 10.2995 | 0.1610 | 0.4636 | 0.0050 | 0.7756 | 2465 | 22 | 2462 | 14 | 2455 | 22 | **2465** | **22** | 99% |
| 81 | 35 | 0.1607 | 0.0056 | 10.3321 | 0.4896 | 0.4626 | 0.0117 | 1.7957 | 2465 | 59 | 2465 | 44 | 2451 | 51 | **2465** | **59** | 99% |
| 82 | 9 | 0.1610 | 0.0013 | 10.3083 | 0.0918 | 0.4637 | 0.0029 | 0.7587 | 2466 | 13 | 2463 | 8 | 2456 | 13 | **2466** | **13** | 99% |
| 83 | 89 | 0.1614 | 0.0018 | 10.3025 | 0.1469 | 0.4629 | 0.0060 | 1.1806 | 2472 | 24 | 2462 | 13 | 2452 | 26 | **2472** | **24** | 99% |
| 84 | 23 | 0.1619 | 0.0027 | 10.3687 | 0.1762 | 0.4649 | 0.0046 | 0.9743 | 2476 | 28 | 2468 | 16 | 2461 | 20 | **2476** | **28** | 99% |
| 85 | 26 | 0.1619 | 0.0020 | 10.2953 | 0.1346 | 0.4607 | 0.0039 | 0.3875 | 2476 | 21 | 2462 | 12 | 2443 | 17 | **2476** | **21** | 99% |
| 86 | 5 | 0.1620 | 0.0035 | 10.3469 | 0.2368 | 0.4642 | 0.0058 | 1.0043 | 2477 | 37 | 2466 | 21 | 2458 | 26 | **2477** | **37** | 99% |
| 87 | 90 | 0.1656 | 0.0019 | 10.8939 | 0.1470 | 0.4775 | 0.0046 | 0.5439 | 2514 | 19 | 2514 | 13 | 2516 | 20 | **2514** | **19** | 99% |
| 88 | 15 | 0.1657 | 0.0020 | 11.0638 | 0.1654 | 0.4835 | 0.0047 | 0.9267 | 2515 | 20 | 2529 | 14 | 2542 | 20 | **2515** | **20** | 99% |
| 89 | 87 | 0.1659 | 0.0014 | 11.0274 | 0.1410 | 0.4808 | 0.0040 | 0.7082 | 2517 | 14 | 2525 | 12 | 2531 | 17 | **2517** | **14** | 99% |
| 90 | 86 | 0.1669 | 0.0047 | 11.0555 | 0.3677 | 0.4808 | 0.0090 | 0.7650 | 2527 | 47 | 2528 | 31 | 2531 | 39 | **2527** | **47** | 99% |
| **Sunjiagou Formation, sample 2015BDSJG-1, GPS: 39° 00′ 13.47″ N, 111° 01′ 01.47″ E** | | | | | | | | | | | | | | | | | |
| 1 | 64 | 0.0511 | 0.0019 | 0.2993 | 0.0109 | 0.0426 | 0.0005 | 0.3736 | 256 | 81 | 266 | 9 | 269 | 3 | **269** | **3** | 98% |
| 2 | 92 | 0.0552 | 0.0053 | 0.3170 | 0.0282 | 0.0428 | 0.0010 | 0.8720 | 417 | 217 | 280 | 22 | 270 | 6 | **270** | **6** | 96% |
| 3 | 94 | 0.0577 | 0.0050 | 0.3342 | 0.0257 | 0.0432 | 0.0009 | 0.5117 | 520 | 191 | 293 | 20 | 272 | 5 | **272** | **5** | 92% |
| ~~4~~ | ~~60~~ | ~~0.0657~~ | ~~0.0035~~ | ~~0.3852~~ | ~~0.0207~~ | ~~0.0433~~ | ~~0.0009~~ | ~~1.2173~~ | ~~798~~ | ~~113~~ | ~~331~~ | ~~15~~ | ~~273~~ | ~~5~~ | **~~273~~** | **~~5~~** | ~~80%~~ |
| 5 | 93 | 0.0532 | 0.0029 | 0.3233 | 0.0187 | 0.0436 | 0.0007 | 1.2829 | 345 | 126 | 284 | 14 | 275 | 5 | **275** | **5** | 96% |
| 6 | 96 | 0.0587 | 0.0052 | 0.3360 | 0.0286 | 0.0443 | 0.0012 | 0.4862 | 567 | 193 | 294 | 22 | 279 | 7 | **279** | **7** | 94% |
| 7 | 1 | 0.0540 | 0.0023 | 0.3588 | 0.0150 | 0.0483 | 0.0007 | 0.7801 | 369 | 94 | 311 | 11 | 304 | 4 | **304** | **4** | 97% |
| 8 | 79 | 0.0565 | 0.0019 | 0.3757 | 0.0120 | 0.0484 | 0.0005 | 0.9105 | 472 | 74 | 324 | 9 | 305 | 3 | **305** | **3** | 93% |
| 9 | 24 | 0.0587 | 0.0039 | 0.3790 | 0.0241 | 0.0486 | 0.0009 | 0.4493 | 554 | 144 | 326 | 18 | 306 | 6 | **306** | **6** | 93% |
| 10 | 30 | 0.0619 | 0.0050 | 0.3966 | 0.0276 | 0.0487 | 0.0010 | 0.6759 | 672 | 168 | 339 | 20 | 306 | 6 | **306** | **6** | 89% |
| 11 | 36 | 0.0568 | 0.0031 | 0.3834 | 0.0216 | 0.0488 | 0.0007 | 0.6790 | 483 | 122 | 330 | 16 | 307 | 4 | **307** | **4** | 92% |
| 12 | 50 | 0.0596 | 0.0054 | 0.3822 | 0.0244 | 0.0492 | 0.0011 | 0.7714 | 587 | 192 | 329 | 18 | 309 | 7 | **309** | **7** | 93% |
| 13 | 84 | 0.0597 | 0.0050 | 0.4232 | 0.0368 | 0.0520 | 0.0021 | 1.3203 | 591 | 183 | 358 | 26 | 327 | 13 | **327** | **13** | 90% |
| 14 | 9 | 0.0534 | 0.0019 | 0.3885 | 0.0129 | 0.0533 | 0.0007 | 0.6977 | 346 | 86 | 333 | 9 | 335 | 4 | **335** | **4** | 99% |
| 15 | 5 | 0.0508 | 0.0026 | 0.3794 | 0.0203 | 0.0541 | 0.0008 | 0.8132 | 232 | 119 | 327 | 15 | 340 | 5 | **340** | **5** | 96% |
| 16 | 59 | 0.0538 | 0.0055 | 0.3901 | 0.0394 | 0.0543 | 0.0021 | 0.8688 | 361 | 233 | 334 | 29 | 341 | 13 | **341** | **13** | 98% |
| 17 | 32 | ~~0.0795~~ | ~~0.0052~~ | ~~0.5871~~ | ~~0.0379~~ | ~~0.0543~~ | ~~0.0013~~ | ~~1.0581~~ | ~~1185~~ | ~~130~~ | ~~469~~ | ~~24~~ | ~~341~~ | ~~8~~ | **~~341~~** | **~~8~~** | ~~68%~~ |
| 18 | 46 | 0.0587 | 0.0037 | 0.4224 | 0.0254 | 0.0545 | 0.0010 | 0.9518 | 554 | 137 | 358 | 18 | 342 | 6 | **342** | **6** | 95% |
| 19 | 22 | 0.0594 | 0.0010 | 0.4455 | 0.0072 | 0.0545 | 0.0003 | 0.2671 | 589 | 37 | 374 | 5 | 342 | 2 | **342** | **2** | 91% |
| 20 | 10 | 0.0563 | 0.0030 | 0.4197 | 0.0195 | 0.0548 | 0.0012 | 0.3602 | 465 | 117 | 356 | 14 | 344 | 7 | **344** | **7** | 96% |
| 21 | 88 | 0.0571 | 0.0043 | 0.4234 | 0.0293 | 0.0549 | 0.0011 | 1.1071 | 494 | 165 | 359 | 21 | 344 | 7 | **344** | **7** | 95% |
| 22 | 6 | 0.0552 | 0.0052 | 0.4127 | 0.0394 | 0.0550 | 0.0020 | 0.8925 | 420 | 211 | 351 | 28 | 345 | 12 | **345** | **12** | 98% |
| 23 | 74 | 0.0604 | 0.0039 | 0.4860 | 0.0244 | 0.0597 | 0.0009 | 1.0196 | 620 | 141 | 402 | 17 | 374 | 6 | **374** | **6** | 92% |
| 24 | 48 | 0.0571 | 0.0019 | 0.4782 | 0.0166 | 0.0606 | 0.0006 | 0.3997 | 494 | 74 | 397 | 11 | 379 | 4 | **379** | **4** | 95% |
| 25 | 90 | 0.0588 | 0.0027 | 0.5231 | 0.0238 | 0.0647 | 0.0008 | 0.3751 | 561 | 102 | 427 | 16 | 404 | 5 | **404** | **5** | 94% |
| 26 | 100 | 0.0561 | 0.0020 | 0.5027 | 0.0179 | 0.0651 | 0.0008 | 0.8424 | 457 | 106 | 414 | 12 | 407 | 5 | **407** | **5** | 98% |
| 27 | 2 | 0.0556 | 0.0038 | 0.4924 | 0.0327 | 0.0656 | 0.0013 | 0.4374 | 435 | 156 | 407 | 22 | 410 | 8 | **410** | **8** | 99% |
| 28 | 34 | 0.0652 | 0.0055 | 0.6325 | 0.0464 | 0.0736 | 0.0014 | 0.4221 | 789 | 178 | 498 | 29 | 458 | 8 | **458** | **8** | 91% |
| ~~29~~ | ~~58~~ | ~~0.1026~~ | ~~0.0058~~ | ~~4.2112~~ | ~~0.2382~~ | ~~0.3008~~ | ~~0.0057~~ | ~~0.6339~~ | ~~1672~~ | ~~104~~ | ~~1676~~ | ~~46~~ | ~~1695~~ | ~~28~~ | **~~1672~~** | **~~104~~** | ~~98%~~ |
| 30 | 95 | 0.1005 | 0.0036 | 4.1611 | 0.1574 | 0.3025 | 0.0056 | 0.5146 | 1635 | 66 | 1666 | 31 | 1704 | 28 | **1635** | **66** | 97% |
| 31 | 12 | 0.1035 | 0.0023 | 4.3171 | 0.0961 | 0.3026 | 0.0032 | 0.5718 | 1689 | 41 | 1697 | 18 | 1704 | 16 | **1689** | **41** | 99% |
| 32 | 28 | 0.1633 | 0.0040 | 11.8052 | 0.3138 | 0.5254 | 0.0081 | 0.4806 | 2490 | 42 | 2589 | 25 | 2722 | 34 | **2490** | **42** | 94% |
| 33 | 45 | 0.1093 | 0.0029 | 4.6649 | 0.1206 | 0.3095 | 0.0035 | 1.3137 | 1789 | 48 | 1761 | 22 | 1738 | 17 | **1789** | **48** | 98% |
| 34 | 99 | 0.1091 | 0.0023 | 4.6976 | 0.1078 | 0.3119 | 0.0038 | 0.6299 | 1784 | 38 | 1767 | 19 | 1750 | 19 | **1784** | **38** | 99% |
| 35 | 40 | 0.1067 | 0.0052 | 4.6501 | 0.2505 | 0.3148 | 0.0063 | 0.6508 | 1744 | 89 | 1758 | 45 | 1764 | 31 | **1744** | **89** | 99% |
| 36 | 8 | 0.1094 | 0.0043 | 4.7267 | 0.1707 | 0.3220 | 0.0062 | 0.8719 | 1791 | 72 | 1772 | 30 | 1799 | 30 | **1791** | **72** | 98% |
| 37 | 35 | 0.1051 | 0.0030 | 4.6858 | 0.1545 | 0.3240 | 0.0074 | 0.8637 | 1716 | 52 | 1765 | 28 | 1809 | 36 | **1716** | **52** | 97% |
| ~~38~~ | ~~78~~ | ~~0.1059~~ | ~~0.0017~~ | ~~3.2345~~ | ~~0.0582~~ | ~~0.2217~~ | ~~0.0029~~ | ~~0.3467~~ | ~~1731~~ | ~~30~~ | ~~1465~~ | ~~14~~ | ~~1291~~ | ~~15~~ | **~~1731~~** | **~~30~~** | ~~87%~~ |
| 39 | 54 | 0.1223 | 0.0017 | 5.4823 | 0.0748 | 0.3247 | 0.0024 | 0.1427 | 1991 | 24 | 1898 | 12 | 1813 | 12 | **1991** | **24** | 95% |
| 40 | 53 | 0.1160 | 0.0031 | 5.2859 | 0.1337 | 0.3327 | 0.0044 | 1.2850 | 1896 | 48 | 1867 | 22 | 1851 | 21 | **1896** | **48** | 99% |
| 41 | 62 | 0.1196 | 0.0033 | 5.5035 | 0.1727 | 0.3328 | 0.0047 | 0.5671 | 1950 | 48 | 1901 | 27 | 1852 | 23 | **1950** | **48** | 97% |
| 42 | 49 | 0.1162 | 0.0024 | 5.3465 | 0.1231 | 0.3330 | 0.0040 | 0.8864 | 1898 | 41 | 1876 | 20 | 1853 | 19 | **1898** | **41** | 98% |
| 43 | 75 | 0.1159 | 0.0028 | 5.3318 | 0.1394 | 0.3334 | 0.0040 | 0.5960 | 1894 | 43 | 1874 | 22 | 1855 | 19 | **1894** | **43** | 98% |
| 44 | 86 | 0.1199 | 0.0055 | 5.4554 | 0.2442 | 0.3338 | 0.0071 | 0.9557 | 1955 | 82 | 1894 | 38 | 1857 | 35 | **1955** | **82** | 98% |
| 45 | 68 | 0.1182 | 0.0018 | 5.4610 | 0.1037 | 0.3344 | 0.0040 | 0.3676 | 1929 | 32 | 1894 | 16 | 1860 | 19 | **1929** | **32** | 98% |
| 46 | 81 | 0.1127 | 0.0032 | 5.2491 | 0.1565 | 0.3370 | 0.0037 | 0.7620 | 1844 | 51 | 1861 | 25 | 1872 | 18 | **1844** | **51** | 99% |
| 47 | 63 | 0.1137 | 0.0036 | 5.2944 | 0.1675 | 0.3376 | 0.0040 | 0.7058 | 1861 | 53 | 1868 | 27 | 1875 | 19 | **1861** | **53** | 99% |
| 48 | 76 | 0.1128 | 0.0017 | 5.2498 | 0.0755 | 0.3382 | 0.0030 | 0.6438 | 1856 | 27 | 1861 | 12 | 1878 | 15 | **1856** | **27** | 99% |
| 49 | 27 | 0.1108 | 0.0022 | 5.1553 | 0.1070 | 0.3384 | 0.0042 | 0.5570 | 1813 | 36 | 1845 | 18 | 1879 | 20 | **1813** | **36** | 98% |
| 50 | 89 | 0.1117 | 0.0019 | 5.2074 | 0.0953 | 0.3386 | 0.0036 | 0.9445 | 1828 | 31 | 1854 | 16 | 1880 | 17 | **1828** | **31** | 98% |
| 51 | 33 | 0.1143 | 0.0055 | 5.4040 | 0.2764 | 0.3413 | 0.0057 | 0.8853 | 1869 | 87 | 1885 | 44 | 1893 | 27 | **1869** | **87** | 99% |
| 52 | 11 | 0.1132 | 0.0057 | 5.2752 | 0.2604 | 0.3437 | 0.0084 | 1.4420 | 1854 | 91 | 1865 | 42 | 1905 | 40 | **1854** | **91** | 97% |
| 53 | 21 | 0.1182 | 0.0025 | 5.5792 | 0.1180 | 0.3438 | 0.0070 | 0.7869 | 1931 | 37 | 1913 | 18 | 1905 | 34 | **1931** | **37** | 99% |
| 54 | 20 | 0.1170 | 0.0032 | 5.5670 | 0.2083 | 0.3438 | 0.0075 | 0.6809 | 1911 | 49 | 1911 | 32 | 1905 | 36 | **1911** | **49** | 99% |
| 55 | 91 | 0.1465 | 0.0014 | 6.9571 | 0.0645 | 0.3441 | 0.0021 | 0.1373 | 2305 | 17 | 2106 | 8 | 1906 | 10 | **2305** | **17** | 90% |
| 56 | 23 | 0.1296 | 0.0027 | 6.7265 | 0.1502 | 0.3771 | 0.0058 | 0.5204 | 2094 | 38 | 2076 | 20 | 2063 | 27 | **2094** | **38** | 99% |
| 57 | 71 | 0.1301 | 0.0039 | 6.8062 | 0.2291 | 0.3793 | 0.0057 | 0.3828 | 2099 | 48 | 2087 | 30 | 2073 | 26 | **2099** | **48** | 99% |
| 58 | 14 | 0.1334 | 0.0028 | 6.9726 | 0.1568 | 0.3799 | 0.0065 | 0.7121 | 2144 | 37 | 2108 | 20 | 2076 | 30 | **2144** | **37** | 98% |
| 59 | 52 | 0.1236 | 0.0025 | 6.5556 | 0.1484 | 0.3839 | 0.0042 | 0.5223 | 2009 | 35 | 2053 | 20 | 2094 | 19 | **2009** | **35** | 98% |
| 60 | 67 | 0.1412 | 0.0061 | 7.8357 | 0.3630 | 0.4060 | 0.0095 | 2.0411 | 2243 | 75 | 2212 | 42 | 2197 | 44 | **2243** | **75** | 99% |
| 61 | 82 | 0.1520 | 0.0020 | 8.9214 | 0.1179 | 0.4261 | 0.0037 | 0.5078 | 2368 | 22 | 2330 | 12 | 2288 | 17 | **2368** | **22** | 98% |
| 62 | 55 | 0.1541 | 0.0051 | 8.9015 | 0.3179 | 0.4289 | 0.0127 | 0.9379 | 2392 | 52 | 2328 | 33 | 2301 | 57 | **2392** | **52** | 98% |
| 63 | 31 | 0.1525 | 0.0039 | 9.0428 | 0.3216 | 0.4309 | 0.0132 | 0.7327 | 2376 | 44 | 2342 | 33 | 2310 | 60 | **2376** | **44** | 98% |
| 64 | 13 | 0.1527 | 0.0017 | 9.1351 | 0.1370 | 0.4333 | 0.0056 | 0.4094 | 2376 | 19 | 2352 | 14 | 2320 | 25 | **2376** | **19** | 98% |
| 65 | 43 | 0.1519 | 0.0034 | 9.0743 | 0.2044 | 0.4335 | 0.0053 | 1.3481 | 2369 | 38 | 2346 | 21 | 2321 | 24 | **2369** | **38** | 98% |
| 66 | 37 | 0.1518 | 0.0032 | 9.0810 | 0.2717 | 0.4341 | 0.0118 | 0.5960 | 2369 | 35 | 2346 | 27 | 2324 | 53 | **2369** | **35** | 99% |
| 67 | 61 | 0.1488 | 0.0033 | 8.9272 | 0.1993 | 0.4343 | 0.0042 | 1.7271 | 2332 | 37 | 2331 | 20 | 2325 | 19 | **2332** | **37** | 99% |
| 68 | 16 | 0.1473 | 0.0052 | 8.8892 | 0.3806 | 0.4356 | 0.0114 | 3.0394 | 2317 | 60 | 2327 | 39 | 2331 | 51 | **2317** | **60** | 99% |
| ~~69~~ | ~~57~~ | ~~0.1574~~ | ~~0.0024~~ | ~~10.0402~~ | ~~0.1678~~ | ~~0.4621~~ | ~~0.0044~~ | ~~0.3723~~ | ~~2427~~ | ~~173~~ | ~~2438~~ | ~~15~~ | ~~2449~~ | ~~19~~ | **~~2427~~** | **~~173~~** | ~~99%~~ |
| 70 | 83 | 0.1624 | 0.0024 | 10.1651 | 0.1558 | 0.4549 | 0.0046 | 0.6128 | 2481 | 25 | 2450 | 14 | 2417 | 20 | **2481** | **25** | 98% |
| 71 | 38 | 0.1609 | 0.0028 | 10.1307 | 0.1989 | 0.4558 | 0.0060 | 0.3889 | 2465 | 29 | 2447 | 18 | 2421 | 27 | **2465** | **29** | 98% |
| 72 | 51 | 0.1619 | 0.0030 | 10.1667 | 0.1887 | 0.4559 | 0.0046 | 1.0252 | 2476 | 31 | 2450 | 17 | 2421 | 20 | **2476** | **31** | 98% |
| 73 | 56 | 0.1597 | 0.0023 | 10.0725 | 0.1561 | 0.4566 | 0.0038 | 0.4711 | 2454 | 24 | 2441 | 14 | 2425 | 17 | **2454** | **24** | 99% |
| 74 | 87 | 0.1594 | 0.0007 | 10.0595 | 0.0547 | 0.4568 | 0.0020 | 0.7113 | 2450 | 8 | 2440 | 5 | 2425 | 9 | **2450** | **8** | 99% |
| 75 | 44 | 0.1609 | 0.0036 | 10.1497 | 0.2179 | 0.4571 | 0.0040 | 1.1106 | 2465 | 37 | 2449 | 20 | 2427 | 18 | **2465** | **37** | 99% |
| 76 | 85 | 0.1611 | 0.0017 | 10.1687 | 0.1266 | 0.4576 | 0.0045 | 0.3784 | 2478 | 17 | 2450 | 12 | 2429 | 20 | **2478** | **17** | 99% |
| 77 | 73 | 0.1599 | 0.0019 | 10.1171 | 0.1453 | 0.4585 | 0.0047 | 0.3716 | 2455 | 21 | 2446 | 13 | 2433 | 21 | **2455** | **21** | 99% |
| 78 | 69 | 0.1587 | 0.0026 | 10.0359 | 0.1677 | 0.4587 | 0.0041 | 0.6440 | 2442 | 28 | 2438 | 15 | 2434 | 18 | **2442** | **28** | 99% |
| 79 | 15 | 0.1584 | 0.0032 | 10.1783 | 0.3286 | 0.4601 | 0.0099 | 0.2380 | 2439 | 34 | 2451 | 30 | 2440 | 44 | **2439** | **34** | 99% |
| 80 | 65 | 0.1605 | 0.0023 | 10.2161 | 0.1512 | 0.4615 | 0.0040 | 0.2878 | 2461 | 24 | 2455 | 14 | 2446 | 18 | **2461** | **24** | 99% |
| 81 | 70 | 0.1596 | 0.0033 | 10.2021 | 0.2409 | 0.4626 | 0.0053 | 0.3413 | 2451 | 35 | 2453 | 22 | 2451 | 23 | **2451** | **35** | 99% |
| 82 | 39 | 0.1565 | 0.0025 | 10.0156 | 0.1556 | 0.4632 | 0.0035 | 0.6278 | 2418 | 27 | 2436 | 14 | 2453 | 15 | **2418** | **27** | 99% |
| 83 | 97 | 0.1579 | 0.0040 | 10.0503 | 0.2488 | 0.4638 | 0.0060 | 1.0818 | 2435 | 43 | 2439 | 23 | 2456 | 26 | **2435** | **43** | 99% |
| 84 | 4 | 0.1570 | 0.0015 | 10.0804 | 0.1754 | 0.4649 | 0.0073 | 0.2941 | 2433 | 16 | 2442 | 16 | 2461 | 32 | **2433** | **16** | 99% |
| 85 | 66 | 0.1572 | 0.0068 | 10.0413 | 0.4324 | 0.4651 | 0.0133 | 0.9473 | 2428 | 73 | 2439 | 40 | 2462 | 58 | **2428** | **73** | 99% |
| 86 | 17 | 0.1551 | 0.0028 | 9.9193 | 0.2147 | 0.4657 | 0.0092 | 0.7820 | 2403 | 31 | 2427 | 20 | 2465 | 40 | **2403** | **31** | 98% |
| 87 | 98 | 0.1555 | 0.0038 | 9.9722 | 0.2782 | 0.4675 | 0.0080 | 0.5184 | 2407 | 43 | 2432 | 26 | 2473 | 35 | **2407** | **43** | 98% |
| 88 | 7 | 0.1585 | 0.0045 | 10.1650 | 0.2863 | 0.4695 | 0.0088 | 0.7356 | 2440 | 48 | 2450 | 26 | 2481 | 39 | **2440** | **48** | 98% |
| 89 | 72 | 0.1693 | 0.0024 | 11.1946 | 0.1645 | 0.4795 | 0.0037 | 0.6575 | 2551 | 24 | 2539 | 14 | 2525 | 16 | **2551** | **24** | 99% |
| 90 | 19 | 0.1662 | 0.0017 | 11.0975 | 0.0975 | 0.4835 | 0.0034 | 0.8399 | 2520 | 18 | 2531 | 8 | 2542 | 15 | **2520** | **18** | 99% |
| 91 | 42 | 0.1657 | 0.0046 | 11.0807 | 0.3065 | 0.4835 | 0.0054 | 1.0576 | 2517 | 47 | 2530 | 26 | 2543 | 24 | **2517** | **47** | 99% |
| 92 | 25 | 0.1674 | 0.0040 | 11.2155 | 0.2712 | 0.4869 | 0.0065 | 0.6743 | 2532 | 40 | 2541 | 23 | 2557 | 28 | **2532** | **40** | 99% |
| 93 | 80 | 0.1668 | 0.0018 | 11.2925 | 0.1906 | 0.4912 | 0.0069 | 0.1247 | 2526 | 19 | 2548 | 16 | 2576 | 30 | **2526** | **19** | 98% |
| 94 | 3 | 0.1649 | 0.0024 | 11.1735 | 0.1997 | 0.4922 | 0.0072 | 0.5900 | 2506 | 25 | 2538 | 17 | 2580 | 31 | **2506** | **25** | 98% |
| 95 | 18 | 0.1652 | 0.0030 | 11.1780 | 0.2666 | 0.4922 | 0.0098 | 0.8679 | 2510 | 30 | 2538 | 22 | 2580 | 42 | **2510** | **30** | 98% |
| 96 | 26 | 0.1661 | 0.0020 | 11.3142 | 0.1669 | 0.4942 | 0.0062 | 0.6236 | 2520 | 20 | 2549 | 14 | 2589 | 27 | **2520** | **20** | 98% |
| 97 | 41 | 0.1646 | 0.0037 | 11.2387 | 0.2484 | 0.4948 | 0.0059 | 0.5779 | 2503 | 38 | 2543 | 21 | 2591 | 26 | **2503** | **38** | 98% |
| 98 | 47 | 0.1657 | 0.0037 | 11.3262 | 0.2230 | 0.4963 | 0.0073 | 0.1620 | 2515 | 37 | 2550 | 18 | 2598 | 31 | **2515** | **37** | 98% |
| 99 | 29 | 0.1621 | 0.0024 | 11.3710 | 0.2073 | 0.5089 | 0.0070 | 0.4173 | 2480 | 25 | 2554 | 17 | 2652 | 30 | **2480** | **25** | 96% |
| 100 | 77 | 0.1666 | 0.0084 | 8.8521 | 0.4496 | 0.4149 | 0.0213 | 1.1631 | 2524 | 85 | 2323 | 46 | 2237 | 97 | **2524** | **85** | 96% |

**References**

1. Ward, P. D. et al. Abrupt and gradual extinction among Late Permian land vertebrates in the Karoo Basin, South Africa. *Science* **307**, 709–714 (2005).

2. Aref ’ev, M. P., Kuleshov, V. N. & Pokrovskii, B. G. Carbon and oxygen isotope composition in upper Permian-lower Triassic terrestrial carbonates of the east European platform: A global ecological crisis against the background of an unstable climate. *Geology* **460**, 11–15 (2015).

3. Retallack, G. J. et al. The Permian-Triassic boundary in Antarctica. *Antarctic Science* **17**, 241–258 (2005).

4. Cao, C. et al. Two episodes of 13C-depletion in organic carbon in the latest Permian: Evidence from the terrestrial sequences in northern Xinjiang, China. *Earth Planet. Sci. Lett.* **270**, 251–257 (2008).

5. Korte, C. & Kozur, H. W. Carbon-isotope stratigraphy across the Permian–Triassic boundary: A review. *J. Asian Earth Sci.* **39**, 215–235 (2010).

6. Wang, Z. Q. & Wang, L. X. Late Permian fossil plants from the lower part of the Shiqianfeng (Shihchienfeng) Group in North China. *Bull. Tianjin. Inst. Geol. Min. Res.* **15**, 1–80 (1986).

7. Zhou, T. S. & Zhou, H. Q. Triassic nonmarine strata and flora of China. *Bull. Chin. Acad. Geol. Sci.* 5, 95–110 (1983).

8. Wang, Z. Q. & Wang, L. X. Late Early Triassic fossil plants from upper part of the Shiqianfeng Group in North China. *Shanxi Geol.* **5**, 97–154 (1990).

9. Chu, D. L. et al. Early Triassic wrinkle structures on land: stressed environments and oases for life. *Sci. Rep.* **5**, 10109 (2015).

10. Wang, W. L. Late Permian conchostracan from the Taohaiyingzi formation in Juudmeng, Innermongo. *Acta Palaeontol. Sin.* **1** (1984).

11. Qu, L. F. The palynological assemblage from the Liujiagou formation of Jiaocheng, *Shanxi. Bull. Chin. Acad. Geol. Sci.* **4**, 83–93 (1982).

12. Chu, D. L. et al. Conchostracans from the Permian-Triassic transition in Weibei area of Shaanxi Province and its biostratigraphic. *Earth Sci.* **43**, 3910–3921 (2018).

13. Shen, Y. B. Occurrence of Permian leaid conchostracans in China and its palaeogeographical significance. *Acta Palaeontol. Sin.* **23**, 505–512 (1984).

14. Liu, S. W. Triassic continental strata and conchostracan faunas in China. *Albertiana* **16**, 27–32 (1995).

1. Institute of Geology, Chinese Academy of Geological Sciences, Beijing 100037, China. ^2^ School of Earth Sciences, University of Bristol, Bristol, BS8 1RJ, UK. ^3^British Geological Survey, Maclean Building, Wallingford OX10 8BB, UK. ^4^College of Earth Sciences, East China University of Science and Technology, Nanchang 330013, Jiangxi, China. ^5^Institute of Electronics, Chinese Academy of Sciences, Suzhou 215123, Jiangsu Province, China. ^6^Shanxi Museum of Geology, Taiyuan 030024, China. Correspondence and requests for materials should be addressed to to Y.L ([liuyongqing@cags.ac.cn](mailto:liuyongqing@cags.ac.cn)). [↑](#footnote-ref-1)
